# Knowledge and Self-Reported Clinical Approaches Regarding Medication-Related Osteonecrosis of the Jaw Among Final-Year Dental Students, General Dentists, and Specialist Dentists: An Exploratory Cross-Sectional Study

**DOI:** 10.3390/healthcare14172819

**Published:** 2026-09-02

**Authors:** İsmail Çapar, Çiğdem Şeker

**Affiliations:** Department of Oral and Maxillofacial Radiology, Faculty of Dentistry, Zonguldak Bülent Ecevit University, 67100 Zonguldak, Turkey; cigdem.seker@beun.edu.tr

**Keywords:** MRONJ, dental education, knowledge, awareness, bisphosphonates

## Abstract

**Background/Objectives:** Medication-related osteonecrosis of the jaw (MRONJ) must be recognized promptly by dental professionals at every career stage, yet whether this recognition differs across professional levels remains unclear. This study compared final-year dental students, general dentists, and specialist dentists in terms of their awareness, pharmacological knowledge, and self-reported clinical approaches to MRONJ. **Methods:** This cross-sectional, exploratory study used a 25-item online questionnaire administered between August and December 2024 to 149 participants (48 students, 51 general dentists, and 50 specialists). Groups were compared using chi-square or Fisher–Freeman–Halton tests, with Benjamini–Hochberg false discovery rate (FDR) correction applied across 79 testable hypotheses; denosumab recognition and routine medication-history questioning were further examined with multivariable logistic regression. Effect sizes were reported as Cramér’s *V*, and regression results were reported as adjusted odds ratios (aORs) with 95% confidence intervals (CIs). **Results:** Raw analyses identified 13 comparisons with *p* < 0.05, of which five remained significant after FDR correction (*q* < 0.05), all involving recognition of bisphosphonate indications and osteonecrosis-associated agents. After FDR correction, no statistically significant group differences were found for general MRONJ awareness, diagnostic criteria, or self-reported clinical approaches (*q* > 0.05), and professional status was not independently associated with denosumab recognition or routine medication-history questioning (*p* > 0.05). **Conclusions:** Responses regarding MRONJ did not follow a consistent knowledge gradient across professional status; the differences that reached statistical significance were confined to specific items of pharmacological knowledge. These findings need confirmation in larger, representative samples, and future research should look more closely at topic-specific educational needs.

## 1. Introduction

Medication-related osteonecrosis of the jaw (MRONJ) is a serious clinical condition linked to antiresorptive and antiangiogenic therapy, marked by persistent, non-healing necrotic lesions of the jaw bones. The American Association of Oral and Maxillofacial Surgeons (AAOMS) defines the condition as exposed necrotic bone, or bone that can be probed through an intraoral or extraoral fistula in the maxillofacial region, persisting for more than eight weeks in a patient with current or previous antiresorptive or antiangiogenic therapy and no history of radiation therapy to the jaws or obvious metastatic jaw disease [1,2]. Early descriptions of jaw osteonecrosis date back to “phossy jaw,” an occupational disease among nineteenth-century match-factory workers chronically exposed to white phosphorus, but the modern concept of medication-related osteonecrosis was introduced only in 2003, when Robert E. Marx and colleagues reported 36 cases linked to bisphosphonate therapy [3]. The condition was initially thought to be specific to bisphosphonates but was later recognized to also occur with denosumab and various antiangiogenic drugs. AAOMS accordingly redefined it as MRONJ in 2014, and its most recent update, in 2022, further refined patient-selection criteria and standardized the condition’s clinical and radiological features [1].

The incidence of MRONJ is low overall but varies with the type, dose, and route of administration of the causative drug: reported rates range from approximately 0.02% to 0.05% among patients receiving low-dose, mainly oral, bisphosphonates for osteoporosis, and are considerably higher among those receiving intravenous or high-dose antiresorptive therapy [2]. In dental practice, MRONJ is therefore encountered most often in patients treated for osteoporosis with oral bisphosphonates, and at substantially higher risk in oncology patients receiving high-dose intravenous antiresorptive therapy for bone metastases of solid tumors or multiple myeloma [2].

The etiology of MRONJ is multifactorial. Local risk factors include periodontal disease, periapical infections, invasive dental procedures, prosthesis-related trauma, poor oral hygiene, and anatomical prominences [4,5]. Pharmacologically, nitrogen-containing, high-potency bisphosphonates such as zoledronate and ibandronate markedly increase MRONJ risk, particularly with long-term use [6]. Systemic factors include malignancy, diabetes mellitus, corticosteroid use, renal insufficiency, vitamin deficiencies, and immunosuppressive states [7,8].

Although the pathophysiology of MRONJ has not been fully elucidated, it is thought to arise from the interplay of suppressed bone remodeling, inflammation and infection, inhibition of angiogenesis, and soft-tissue toxicity. Suppression of osteoclast activity by antiresorptive agents may impair the repair of microdamage in jaw bones, which normally undergo high rates of turnover [5]. The frequent detection of *Actinomyces* species supports a possible infectious contribution [9], while the adverse effects of vascular endothelial growth factor (VEGF) inhibitors and bisphosphonates on tissue healing may further facilitate the osteonecrotic process [10].

Clinically, MRONJ presents with a broad spectrum of findings. The most common finding is exposed necrotic bone (approximately 94% of cases), frequently accompanied by pain, mucosal inflammation, fistula formation, sinus involvement, neurosensory disturbances, and tooth mobility [4]. Although biochemical markers such as serum C-terminal telopeptide (CTX) levels can provide information about bone turnover, the diagnosis of MRONJ relies primarily on clinical criteria [3,11]. The staging system defined by the AAOMS ranges from patients “at risk”—those receiving antiresorptive therapy without clinical evidence of necrotic bone—through Stages 0–3. Stage 1 is characterized by asymptomatic exposed necrotic bone or a probe-able fistula, and Stage 2 by infection and pain. Stage 3 comprises more advanced disease extending beyond the alveolar bone, which may lead to complications such as pathological fracture, extraoral fistula, or oroantral/oronasal communication [2].

Prevention and early diagnosis of MRONJ depend on the appropriate assessment of at-risk patients and careful planning of invasive dental procedures. In this process, it is essential that dentists recognize antiresorptive and antiangiogenic therapies, their associated risk factors, and early clinical signs [2]. Identifying knowledge gaps at different educational and professional stages may therefore contribute to the development of more targeted educational content.

The aim of this study was to compare the awareness, pharmacological knowledge, and self-reported clinical approaches regarding MRONJ among final-year dental students, general dentists, and specialist dentists, and to exploratively evaluate the knowledge patterns that may be observed across different educational and professional stages. As the study was exploratory, no single confirmatory primary outcome was defined; nevertheless, it was hypothesized that greater clinical experience and specialization would be associated with higher MRONJ-related knowledge, with specialist dentists expected to show the highest level of knowledge, followed by general dentists and then final-year students.

## 2. Materials and Methods

### 2.1. Study Design and Participants

**Figure 1:**
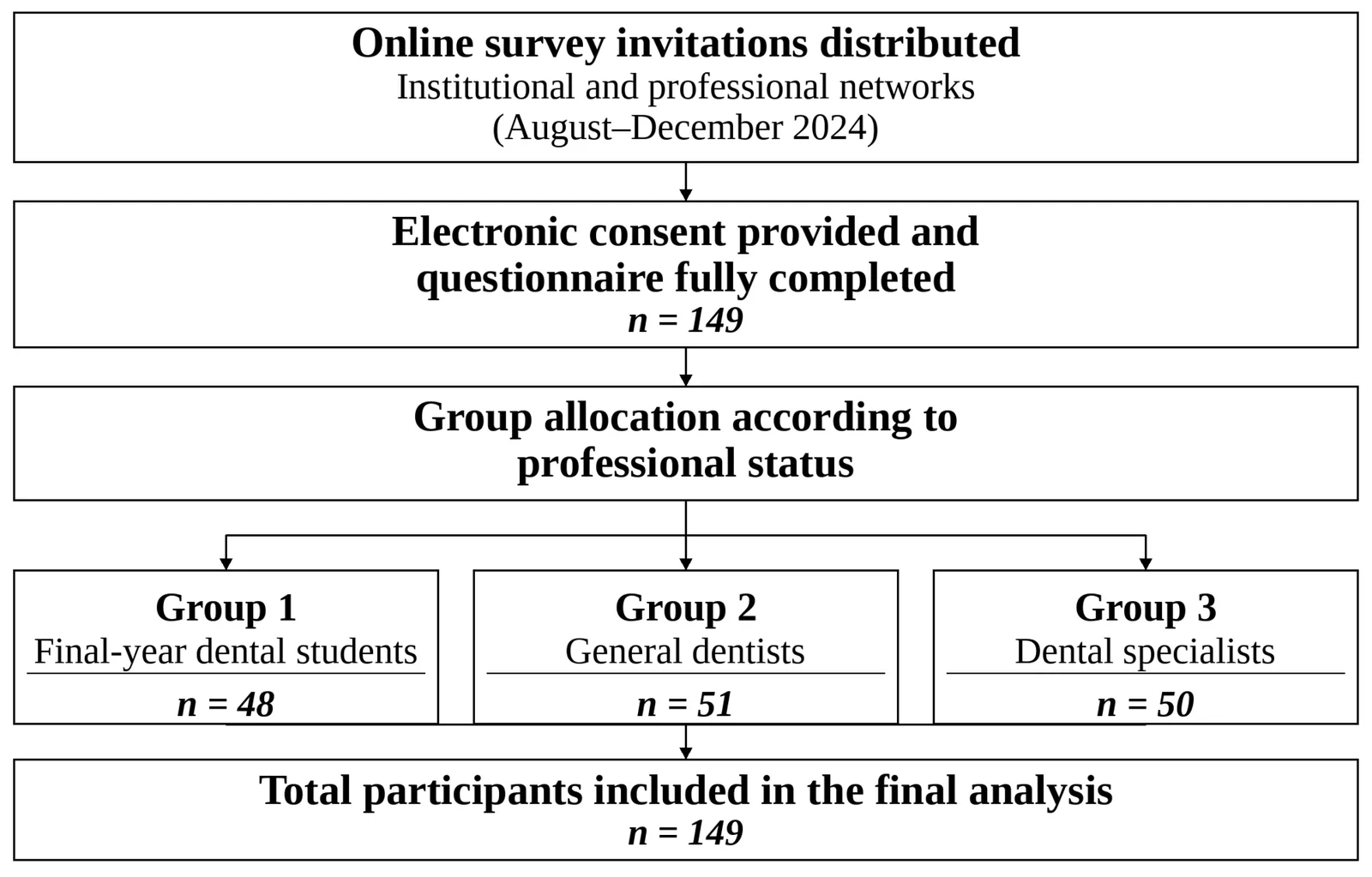
Recruitment, group allocation, and inclusion of participants in the final analysis.

This cross-sectional study was conducted between August and December 2024 using a 25-item online questionnaire (Google Forms; Google LLC, Mountain View, CA, USA) developed from a literature review and expert opinion. Recruitment relied on a large-scale strategy spanning institutional and professional communication channels across Turkey. A standardized study invitation and the questionnaire link were sent through official correspondence to faculty members at dental schools throughout Turkey and to provincial health directorates: faculty members were asked to forward the link to eligible final-year students, and provincial health directorates were asked to forward the link to general and specialist dentists. General and specialist dentists were additionally reached through WhatsApp (Meta Platforms, Inc., Menlo Park, CA, USA), e-mail, and the researchers’ personal and professional networks. The questionnaire link was re-shared through the same channels approximately 10 days after the initial invitation as a reminder. Participation was voluntary, and a non-probability convenience sampling method was used. Figure 1 shows the recruitment of participants, their allocation to groups by professional status, and their inclusion in the final analysis.

### 2.2. Participation and Data Completeness

Volunteers who identified themselves as a final-year dental student, general dentist, or specialist dentist, and who gave electronic informed consent, were included in the study and directed to the questionnaire items. All items in Google Forms were mandatory, so only fully completed questionnaires could be submitted and recorded by the system; consequently, data completeness was ensured for every questionnaire included in the final analysis, and no missing-data imputation or exclusion for missing data was required. Since the system recorded only fully submitted responses, however, neither the partial responses of individuals who began but did not finish the questionnaire, nor the number and characteristics of such individuals, could be captured.

As the questionnaire link could be re-shared through institutional intermediaries and professional networks, an exact denominator for the total number of individuals reached could not be established, and a response rate could therefore not be calculated. The questionnaire also did not collect institutional or provincial information, so the distribution of participants could not be classified by center or geographic region.

### 2.3. Final Sample Composition

All 149 participants who provided electronic informed consent and completed the questionnaire in full were included in the final analysis. The final sample comprised 48 final-year dental students, 51 general dentists, and 50 specialist dentists.

The specialist dentist group spanned eight different dental specialties, with the distribution across specialties presented descriptively in Table 1. Because the study’s primary comparison was planned across professional-status groups, specialists were treated as a single combined group in the main analyses. As the number of participants per specialty was small (*n* = 4–8) and expected cell counts were insufficient for some response categories, no inferential statistical comparisons were performed among individual specialties.

### 2.4. Sample Size Calculation

As this study did not have a single predetermined primary outcome, analyses of the questionnaire items were treated as exploratory and hypothesis-generating. An a priori sample size calculation, performed using G*Power version 3.1.9.6 (Heinrich Heine University Düsseldorf, Düsseldorf, Germany), was used to set a recruitment target. A planning effect size of *w* = 0.377 was adopted, based on the magnitude of between-group differences in MRONJ awareness reported by Ekmekçioğlu et al. [12]; for a chi-square test comparing three groups, the degrees of freedom were set at 2, the Type I error rate at 0.05, and statistical power at 95%. G*Power returned a minimum required total sample size of 109, which was rounded up to 111 to ensure a balanced number of participants across groups (at least 37 per group). Allowing for a potential participation loss of approximately 20%, the target sample size was calculated as 111/0.80 = 138.75 and rounded up to 139; the study was ultimately completed with 149 participants. This calculation served as a planning benchmark for the overall recruitment target rather than a confirmatory power estimate for individual questionnaire items.

### 2.5. Questionnaire Structure and Development

The questionnaire comprised four demographic items (Q1–Q4), recording age, sex, educational/professional status (with specialty recorded for specialist dentists), and years of clinical experience, plus 21 core items (Q5–Q25) assessing MRONJ awareness, knowledge, and self-reported clinical approaches, for 25 numbered items in total. The conditional sub-item on specialty was treated as part of the professional-status item (Q3) rather than counted separately. The English version of the questionnaire appears in File S1. Items were developed by reviewing the AAOMS’s current MRONJ diagnosis and management recommendations, the relevant literature, and previously published studies on MRONJ knowledge and awareness and were designed to cover MRONJ diagnostic criteria, associated drugs and their therapeutic indications, risk factors, clinical and radiological findings, and dental treatment approaches. Items were closed-ended, permitting either a single response or the selection of multiple options.

### 2.6. Content Validity and Pilot Testing

The content validity of the draft questionnaire was evaluated by two experts in Oral and Maxillofacial Radiology with 3 and 8 years of professional experience, respectively. They reviewed the items for scope, clinical relevance, clarity, and comprehensibility, and the questionnaire was finalized after incorporating their revisions. Because this evaluation was qualitative, a numerical Content Validity Index was not calculated.

The draft questionnaire was then administered to a pilot group of 30 individuals independent of the final study sample: 10 final-year dental students, 7 general dentists, and 13 specialist dentists. Face validity was assessed with a five-item evaluation form, and pilot participants rated the questionnaire as clear and comprehensible. Based on their feedback, the phrase “continuation of Q19” was removed from Q20 and Q21, and the questionnaire was finalized once this and all other necessary revisions had been made.

### 2.7. Test–Retest Reliability

To evaluate test–retest reliability, the final, unmodified version of the questionnaire was administered to the same pilot group on two occasions, two weeks apart, with no changes to the content, item wording, or response options between administrations. Given the categorical nature of the items, agreement between the two administrations was assessed using Cohen’s kappa (κ) coefficient and the observed percentage of exact agreement: for single-response items, the selected response category was used, whereas for multiple-response items the complete pattern of selected options was used. Spearman’s rank correlation coefficient (ρ) was calculated as a supplementary association measure, using coded responses for single-response items and the number of selected options for multiple-response items; the number of selected options was not used as a knowledge or accuracy score. Cohen’s κ coefficients were interpreted according to the classification of Landis and Koch, and Spearman’s ρ coefficients were interpreted according to that of Schober et al. [13,14], with item-level coefficients and exact agreement rates presented in Table S1. As the questionnaire was designed to capture several distinct domains of MRONJ knowledge and clinical approach rather than a single latent construct, it was not treated as a unidimensional scale and no total score was calculated; internal-consistency indices such as Cronbach’s alpha and the intraclass correlation coefficient (ICC) were therefore not appropriate and were not computed. Reliability was instead evaluated at the item level using Cohen’s κ and Spearman’s ρ. Data from the pilot group were not included in the final study analyses.

### 2.8. Response Coding

For items permitting multiple responses, each option was coded as a separate binary variable (selected = 1, not selected = 0). No item-level total correct score or partial-credit scoring based on the joint selection of all correct options was applied to these items. Analyses were instead based on the frequency and percentage with which each option was selected within participant groups. For knowledge items, the options considered correct were predetermined on the basis of the current literature and expert opinion prior to analysis. Single-response items were analyzed according to the response category selected by each participant.

### 2.9. Ethical Approval and Informed Consent

Ethical approval for this study was obtained from the Non-Interventional Clinical Research Ethics Committee of Zonguldak Bülent Ecevit University (Decision No: 2024/13; Date: 10 July 2024). All procedures were conducted in accordance with the Declaration of Helsinki and its subsequent amendments. Electronic informed consent was obtained from all participants prior to their inclusion in the study.

### 2.10. Statistical Analysis

Categorical variables are presented as number and percentage [*n* (%)]. Group comparisons for the questionnaire items spanning Q5–Q25 were regarded as exploratory and hypothesis-generating. For single-response items, the overall distribution of response categories was compared between groups; for multiple-response items, each option was compared between groups as a separate binary “selected/not selected” variable. The Pearson chi-square test was used, with the Fisher–Freeman–Halton exact test applied when the assumptions of the chi-square test were not met. Effect sizes were expressed using Cramér’s *V*. Values were interpreted using Cohen’s conventional benchmarks (small ≈ 0.10, medium ≈ 0.30, large ≈ 0.50) for a contingency table whose smaller dimension has one degree of freedom, as applied here to the binary between-group comparisons [15].

Benjamini–Hochberg false discovery rate (FDR) correction was applied across a total of 79 testable hypotheses, with raw probability values denoted *p*, FDR-adjusted values denoted *q*, and *q* < 0.05 considered statistically significant. Comparisons that remained significant after FDR correction were followed by Bonferroni-corrected pairwise comparisons. For these pairwise comparisons, the significance threshold was Bonferroni-adjusted for the three possible between-group contrasts (α = 0.05/3 ≈ 0.017). As a post hoc sensitivity analysis conducted in response to reviewer feedback, the comparisons that survived FDR correction, together with denosumab recognition and routine history-taking, were re-examined between general dentists and specialists only (excluding students) using Fisher’s exact test, with Benjamini–Hochberg correction applied across this set of comparisons.

Two separate multivariable binary logistic regression models were constructed: one for the selection of denosumab as an MRONJ-associated agent and one for routine questioning of antiresorptive/antiangiogenic drug use in the medical history. The main explanatory variable in both models was professional status, with models adjusted for age group and sex; age was categorized as <25, 25–34, and ≥35 years. Given the structural overlap between professional status and clinical experience, the latter was not included in the primary models. Results are presented as adjusted odds ratios (aORs) with 95% confidence intervals (CIs) and *p*-values. All tests were two-tailed, and analyses were performed using IBM SPSS Statistics, version 27.0 (IBM Corp., Armonk, NY, USA).

## 3. Results

Of the 149 participants, 32.2% (*n* = 48) were final-year dental students, 34.2% (*n* = 51) were general dentists, and 33.6% (*n* = 50) were specialist dentists. Most were aged 25–34 years (45.6%), followed by those under 25 (36.9%), and men accounted for 65.8% of the sample. Final-year students made up 32.2% of participants, and 17.4% of the full sample had more than 10 years of professional experience. Within the specialist group, Oral and Maxillofacial Surgery and Endodontics were each represented by eight participants (16%); Oral and Maxillofacial Radiology, Periodontology, Prosthodontics, Restorative Dentistry, and Pediatric Dentistry by six each (12%); and Orthodontics by four (8%) (Table 1). A descriptive breakdown of the key MRONJ knowledge and clinical-approach items across the eight individual specialties is shown in Table S2; given the small number of participants per specialty, these data are reported descriptively (*n* and %) without inferential comparisons.

The content validity of the questionnaire was evaluated by two experts. In the test–retest assessment, exact agreement rates ranged from 46.7% to 96.7%, Cohen’s κ coefficients ranged from 0.408 to 0.874, and Spearman’s ρ coefficients ranged from 0.648 to 0.920. According to the Spearman ρ values, Q8, Q15, Q21, and Q23 showed moderate correlation; Q6 and Q7 showed very strong correlation; and the remaining items showed strong correlation. Item-level reliability results are presented in Table S1.

Group-based response distributions and item-level comparisons for all 21 items spanning Q5–Q25 are presented in File S2. The tables in the main text are limited to the key findings directly related to the aims of the study.

Of the 79 testable hypotheses, 13 showed a group difference at the raw *p* < 0.05 level; after Benjamini–Hochberg FDR correction, five comparisons remained significant.

Groups reported comparable general awareness of the MRONJ concept (*p* = 0.696; *q* = 0.839). When asked to identify the AAOMS diagnostic criteria, raw analyses suggested some between-group variation for a history of antiresorptive/antiangiogenic drug use and for exposed bone or a probe-able fistula persisting more than eight weeks; however, neither difference survived FDR correction (*q* = 0.243 and *q* = 0.259, respectively) (Table 2).

Self-assessed knowledge of antiresorptive and antiangiogenic drugs was similarly comparable across groups (*p* = 0.144; *q* = 0.474). All students named university coursework as their first source of information, whereas specialists more often cited scientific journals; again, neither pattern held up after FDR correction (*q* = 0.072 and *q* = 0.079, respectively). A similar picture emerged for recognizing antiresorptive agents as a drug class linked to osteonecrosis: the raw difference was significant (*p* = 0.006) but did not survive correction (*q* = 0.072) (Table 2).

The five comparisons that did survive FDR correction all involved pharmacological knowledge. Specialists more often recognized bone metastases of solid tumors as a bisphosphonate indication than students or general dentists (*q* = 0.044; V = 0.282), and both general dentists and specialists outperformed students in recognizing multiple myeloma as an indication (*q* = 0.044; V = 0.281). By the prespecified benchmarks, both effects were small (Table 2).

Recognition of individual osteonecrosis-associated agents followed a similar gradient: alendronate was identified by 18.8% of students, 45.1% of general dentists, and 58.0% of specialists (*q* = 0.012; V = 0.329); pamidronate by 22.9%, 47.1%, and 58.0% (*q* = 0.043; V = 0.293); and zoledronate by 35.4%, 62.7%, and 80.0% (*q* = 0.003; V = 0.371). Effect sizes were moderate for alendronate and zoledronate and small for pamidronate. Denosumab showed the opposite pattern, being selected more often by students than by the other two groups (79.2% vs. 51.0% and 54.0%), but this difference did not survive FDR correction (*p* = 0.007; *q* = 0.072) (Table 2).

For the two self-reported clinical-approach items, routine questioning about antiresorptive or antiangiogenic drug use was reported by 70.8% of students, 43.1% of general dentists, and 52.0% of specialists—a raw difference that did not hold after FDR correction (*p* = 0.019; *q* = 0.149). The same was true for basing all treatment decisions on medical consultation (*p* = 0.041; *q* = 0.259) (Table 2).

In the post hoc sensitivity analysis restricted to practicing dentists, recognition of bone metastases of solid tumors as an indication for bisphosphonate therapy was higher among specialists than general dentists (84.0% vs. 54.9%; Fisher’s exact *p* = 0.002; BH-adjusted *q* = 0.016). None of the remaining six comparisons was statistically significant after correction (all *q* ≥ 0.273).

After adjustment for age group and sex, professional status showed no overall effect on selection of denosumab (likelihood-ratio *p* = 0.366) or on routine medication-history questioning (likelihood-ratio *p* = 0.140). The wide confidence intervals around several estimates indicated limited precision; therefore, the models do not support a definitive professional-status gradient for either outcome (Table 3).

## 4. Discussion

This study compared MRONJ-related knowledge and awareness among students, general dentists, and specialist dentists at the item level. After FDR correction across 79 hypotheses, only five group differences retained statistical significance, all relating to recognition of the therapeutic indications of bisphosphonates and of osteonecrosis-associated active substances. No significant group differences emerged, after correction, in general MRONJ awareness, self-assessed knowledge, diagnostic criteria, information sources, or self-reported clinical approaches. This does not mean the groups are equivalent in these domains; it means only that the statistically supported differences in this sample were confined to specific items of pharmacological knowledge, and the findings should be read as item-level differences rather than an overall ranking of knowledge across professional groups. Current guidance does not assess MRONJ risk from the drug name alone, but from the treatment indication, dose, frequency and route of administration, and duration of treatment considered jointly [2]. The significant differences, specifically, were confined to recognition of bone metastases of solid tumors and multiple myeloma as bisphosphonate indications and of alendronate, pamidronate, and zoledronate as osteonecrosis-associated agents, with recognition lowest among students in every case.

Bone metastases of solid tumors were selected as a bisphosphonate indication more often by specialist dentists than by students and general dentists. Recognition rates for multiple myeloma, together with alendronate, pamidronate, and zoledronate, were likewise higher among general dentists and specialists than among students. The most pronounced group difference was observed for zoledronate, which was selected by 80.0% of specialists. Ekmekçioğlu et al. reported that alendronate was recognized by 23.8% of general dentists and 55.6% of specialists, and zoledronate by 22.9% and 58.3%, respectively [12]. This pattern, consistent across both studies, suggests that specialists are more familiar with bisphosphonates that have been in clinical use for longer. Given the cross-sectional design, however, and the fact that potential confounders were not modeled separately, these differences cannot be attributed solely to specialty training or clinical experience.

Specialist status was not associated with higher knowledge across all knowledge domains. Self-assessed knowledge of antiresorptive and antiangiogenic agents did not differ between groups, and most participants rated their knowledge as “some knowledge.” Alhussain et al. similarly reported variability in dentists’ knowledge, practices, and opinions regarding the dental management of patients receiving bisphosphonates [16]. Self-assessed awareness, however, is not equivalent to accurate knowledge of specific drugs, risk factors, and management principles. Participants’ perceived knowledge and their item-level responses should therefore be evaluated separately. Several factors could explain why knowledge did not rise consistently with professional seniority. MRONJ-related pharmacology and risk assessment have entered undergraduate and specialty curricula relatively recently and unevenly, so recent graduates and final-year students may in some respects have been exposed to this content more recently than their more experienced colleagues. MRONJ is also seen infrequently in routine dental practice, meaning that sustained familiarity may depend more on continuing education and clinical setting than on professional title or years of experience. The heterogeneity of the specialist group, which included specialties with little direct involvement in MRONJ management, and the possible self-selection of more interested or knowledgeable volunteers, may have further flattened any gradient. Larger, representative samples would be needed to test these explanations directly.

Denosumab was selected as an osteonecrosis-associated agent by 79.2% of students, 51.0% of general dentists, and 54.0% of specialists. This raw difference was not significant after FDR correction, and the adjusted logistic regression likewise showed no significant independent association between professional status and denosumab selection. The wide confidence intervals in the model reflect considerable uncertainty in the estimates, so students’ knowledge of denosumab cannot be concluded to exceed that of the other groups. Denosumab acts through inhibition of receptor activator of nuclear factor-κB ligand (RANKL), does not accumulate in bone, and behaves differently from bisphosphonates after discontinuation, so distinguishing between these agents remains clinically important for risk assessment [17,18]. Recognition was also consistently higher for long-established nitrogen-containing bisphosphonates, particularly zoledronate, than for denosumab, even though denosumab has been in clinical use for more than a decade. The gap likely traces to the longer presence of bisphosphonates in dental curricula and clinical discourse; awareness of denosumab as an MRONJ-associated agent seems less uniformly established across professional groups.

Poor oral hygiene, periodontal disease, and tobacco use were frequently selected across all groups, whereas responses on systemic comorbidities and certain pharmacological risk components were more variable. Exposed bone was recognized by the large majority of participants, but radiographic changes such as lamina dura thickening and widening of the periodontal ligament space were selected less often, pointing to lower familiarity with imaging features that can accompany MRONJ without being specific to it. These radiographic changes are not diagnostic in isolation, however, so their low selection rate should not be read as an inability to recognize early MRONJ cases; nor is this pattern specific to any one professional group, since the group difference for these items was not significant after FDR correction. Responses on interpreting serum CTX values and on a six-month drug holiday from antiresorptive therapy were similarly dispersed, showing no shared approach among participants. Serum CTX alone is not a reliable predictor of MRONJ risk, and decisions on drug discontinuation should not be routine but should instead weigh the treatment indication, the specific agent used, and the individual patient’s risk profile [19,20].

In the surgical scenario, most participants preferred to avoid extraction in a patient who had been taking oral alendronate for osteoporosis for one year, alongside preferences for medical consultation, antibiotic prophylaxis, and CTX assessment; together, these point to a cautious risk perception in this patient group. Avoiding extraction alone, however, does not by itself represent clinical management consistent with current recommendations, and medical consultation, antibiotic use, and CTX assessment cannot be regarded as equivalent protective measures backed by the same level of evidence. Previous survey studies have likewise reported a tendency among students and dentists to avoid surgical procedures, seek consultation, and adopt extra precautions in patients at risk of MRONJ [21,22,23,24]. These findings raise the possibility of a cautious approach, an overestimation of risk, or uncertainty about current management recommendations; in addition, preferences reported in a survey do not necessarily reflect actual clinical behavior or adherence to guidelines. For a patient who has been taking low-dose oral alendronate for one year, current recommendations do not contraindicate necessary extractions, so a blanket preference to avoid extraction may reflect an overestimation of risk rather than management aligned with current guidance [2].

Participants frequently selected preventive approaches, such as extracting residual roots and favoring endodontic treatment in suitable teeth before starting high-dose intravenous bisphosphonate therapy, pointing to general familiarity with dental optimization principles aimed at reducing the need for invasive procedures after treatment [25,26]. At the same time, “I don’t know” responses for some options and variability in treatment-planning preferences show that these principles were not applied uniformly by all participants. As preferences expressed in a multiple-choice survey may not mirror actual clinical decision-making, these results should not be taken as direct evidence of practical competence.

In previous student studies conducted in Turkey, denosumab awareness was reported as 19.6% by Erdil et al. and 8.9% by Şahin et al., and Gaş et al. similarly found that students’ knowledge of MRONJ-related drugs and detailed diagnostic features was limited [27,28,29]. The higher denosumab selection rate among students in the present study may relate to question format, the presentation of response options, sample characteristics, or educational content and should not be read as direct evidence of a temporal increase in knowledge; since the difference was not confirmed by FDR correction or the multivariable analysis, it is best regarded as a descriptive pattern. Likewise, although a numerically higher proportion of students reported questioning antiresorptive or antiangiogenic drug use in the routine history, this was not confirmed in the adjusted analyses. The discrepancy may partly reflect a difference in what was measured: previous student studies have largely assessed the perceived importance of medication history, whereas the present study asked about reported routine practice.

Much of the previous literature [26,27,28,29] evaluated students or dentists in separate samples. Here, comparing students, general dentists, and dentists from different specialties within the same sample, correcting item-level multiple comparisons with the FDR method, and examining selected outcomes with multivariable models together allowed for a more cautious evaluation of apparent group differences. Indeed, only five of the 13 comparisons significant in the raw analyses remained so after FDR correction, showing that interpretations based on uncorrected *p*-values alone can be misleading in studies examining many questionnaire items. Consistent with this, a sensitivity analysis excluding students showed that, among practicing dentists, only recognition of bone metastases as a bisphosphonate indication distinguished specialists from general dentists; the remaining pharmacological differences were no longer significant, suggesting that much of the apparent group variation was driven by lower recognition among students rather than by a broad specialist advantage.

These findings suggest that future educational-needs assessments could address the pharmacological classes and therapeutic indications of relevant drugs, individualized risk assessment, the limitations of radiographic findings, the interpretation of CTX, and current approaches to drug discontinuation. As MRONJ occurs relatively infrequently, particularly in patients on low-dose oral therapy, in-depth and continuously updated knowledge is arguably most needed by the clinicians most likely to manage at-risk or affected patients, such as oral and maxillofacial surgeons and orthodontists treating adult patients, while a sound working knowledge of risk recognition and timely referral remains relevant to all dental professionals. This cross-sectional, exploratory study cannot, however, directly show that any specific educational intervention is necessary or effective. Future research should also incorporate longitudinal and qualitative designs and assess the cost-effectiveness of targeted MRONJ educational interventions.

### Limitations

This study has several limitations. Its cross-sectional design means that causal conclusions cannot be drawn from the associations observed, and reliance on self-reported data may have introduced recall and social desirability bias. The study assessed reported awareness, pharmacological knowledge, and clinical approaches rather than actual clinical behavior, patient records, or patient outcomes, so the extent to which questionnaire responses reflect actual clinical practice cannot be determined.

The voluntary, online nature of participation and the use of non-probability sampling create a risk of selection and self-selection bias. Since the total number of individuals reached, those who declined to participate, and those who began but did not complete the questionnaire were not recorded, the response rate could not be calculated and non-response bias could not be assessed. Institutional and provincial information was also not collected, so the geographic distribution of the sample and the contribution of different centers could not be determined, and the lack of information on the faculties where student participants were enrolled likewise precluded any examination of inter-institutional differences in curricula and MRONJ-related education. The sample should therefore not be considered nationally representative, and the findings should not be generalized directly to all dental students and dentists in Turkey.

The questionnaire was developed on the basis of a literature review and expert opinion, its content validity was evaluated by two experts, and its test–retest reliability was examined. For six predominantly multiple-response items (Q11, Q12, Q17, Q21, Q23, and Q25), test–retest agreement was only moderate (Cohen’s κ = 0.408–0.549). As κ for multiple-response items was calculated on the complete pattern of selected options, a change in any single option between the two administrations lowered the coefficient, even though the corresponding ordinal associations (Spearman’s ρ) remained mostly strong for these items. This pattern suggests that responses to some detailed, multi-component items were less stable over time, possibly reflecting genuine uncertainty or guessing about specific pharmacological and clinical details, so the related item-level findings should be interpreted with additional caution. Comprehensive psychometric validation of the instrument in larger, independent samples was not performed, however, and the moderate test–retest agreement observed for some items, together with the option-based structure of the questionnaire, may have limited measurement precision and the extent to which participants’ depth of knowledge and clinical reasoning could be assessed.

The specialist dentist group comprised eight different specialties that were not equally represented, which increased its heterogeneity; as the number of participants within each specialty was low, specialty-specific comparisons could not be performed. Treating specialists as a single combined group may have masked differences in how often clinicians in each specialty encounter MRONJ clinically and in their educational background. The findings for the specialist group should therefore not be generalized to any individual specialty. MRONJ is, in particular, a largely surgically oriented condition, so including specialties with limited direct involvement in its diagnosis and management (for example, pediatric dentistry or orthodontics) alongside oral and maxillofacial surgery may have diluted the apparent knowledge level of the combined specialist group; established specialists, moreover, tend to concentrate on their own field and may follow developments outside it less closely. The small number of participants per specialty further limits the certainty of any specialty-specific conclusions, and larger studies with more balanced, ideally probability-based sampling across specialties are needed to confirm these observations.

Although FDR correction was applied across 79 hypotheses to reduce the risk of false positives from multiple comparisons, the analyses were not based on a single predetermined primary outcome and remain exploratory in nature. FDR correction and the limited sample size may have reduced the power to detect some true differences, so non-significant corrected results should not be read as evidence of equivalence between groups. The close relationship among professional status, age, and clinical experience also meant that clinical experience could not be included in the primary regression models, and modeling age using broad categories may have lost some age-related information. The wide confidence intervals in the regression estimates reflect limited precision in determining independent associations.

## 5. Conclusions

This study shows that knowledge and self-reported clinical approaches regarding MRONJ did not increase consistently from students toward general and specialist dentists. After correction for multiple comparisons, differences among professional groups were confined to recognition of specific bisphosphonates and their therapeutic indications, and specialist status carried no consistent advantage in other domains of knowledge or clinical approach; the differences observed in denosumab recognition and routine medication-history questioning were likewise not confirmed by the corrected analyses. In this sample, statistically supported between-group differences were limited to specific pharmacological knowledge items, and no consistent professional-status gradient was demonstrated; future educational initiatives may accordingly be better planned around these identified, topic-specific knowledge needs than around professional status alone. As these findings come from a self-reported, non-representative sample, though, they will need confirmation in larger, more representative cohorts.

## Figures and Tables

**Table 1:** Demographic and professional characteristics of participants.

Variable		*n*	%
Age	<25	55	36.9%
	25–34	68	45.6%
	35–50	23	15.4%
	>50	3	2.0%
Sex	Female	51	34.2%
	Male	98	65.8%
Professional Status	Student	48	32.2%
	General Dentist	51	34.2%
	Dental Specialist	50	33.6%
Specialty	No specialty	99	66.4%
	Oral and Maxillofacial Radiology	6	4.0%
	Oral and Maxillofacial Surgery	8	5.4%
	Endodontics	8	5.4%
	Prosthodontics	6	4.0%
	Restorative Dentistry	6	4.0%
	Periodontology	6	4.0%
	Pediatric Dentistry	6	4.0%
	Orthodontics	4	2.7%
Clinical Experience	Final-year student	48	32.2%
	1–3 years	29	19.5%
	3–5 years	18	12.1%
	5–10 years	28	18.8%
	>10 years	26	17.4%

*n*: number of participants; %: percentage within group. The “No specialty” category comprises all participants without a dental specialty, that is, the final-year dental students (*n* = 48) and general dentists (*n* = 51) combined (*n* = 99).

**Table 2:** Exploratory item-level group comparisons with nominal statistical significance (raw *p* < 0.05).

Question/Response	Student (*n* = 48)	General Dentist (*n* = 51)	Specialist (*n* = 50)	*p*	*q*	*V*
**AAOMS diagnostic criteria**						
History of antiresorptive/antiangiogenic drug use	37 (77.1%)	27 (52.9%)	29 (58.0%)	0.034	0.243	0.213
Exposed bone or probe-able fistula persisting >8 weeks	37 (77.1%)	33 (64.7%)	43 (86.0%)	0.043	0.259	0.206
**Initial information sources for antiresorptive/antiangiogenic drugs**						
University	48 (100%)	41 (80.4%)	43 (86.0%)	0.007	0.072	0.258
Scientific journals	5 (10.4%)	5 (9.8%)	15 (30.0%)	0.009	0.079	0.252
**Drug class associated with osteonecrosis**						
Antiresorptive agents	21 (43.8%)	10 (19.6%)	24 (48.0%)	0.006	0.072	0.261
**Bisphosphonate indications**						
Bone metastases of solid tumors	27 (56.3%) ^b^	28 (54.9%) ^b^	42 (84.0%) ^a^	0.003	**0.044**	0.282
Multiple myeloma	8 (16.7%) ^b^	23 (45.1%) ^a^	23 (46.0%) ^a^	0.003	**0.044**	0.281
**Osteonecrosis-associated active substances**						
Alendronate	9 (18.8%) ^b^	23 (45.1%) ^a^	29 (58.0%) ^a^	<0.001	**0.012**	0.329
Pamidronate	11 (22.9%) ^b^	24 (47.1%) ^a^	29 (58.0%) ^a^	0.002	**0.043**	0.293
Zoledronate	17 (35.4%) ^b^	32 (62.7%) ^a^	40 (80.0%) ^a^	<0.001	**0.003**	0.371
Denosumab	38 (79.2%)	26 (51.0%)	27 (54.0%)	0.007	0.072	0.257
**Self-reported clinical approaches**						
Routine questioning of relevant medication history	34 (70.8%)	22 (43.1%)	26 (52.0%)	0.019	0.149	0.231
All treatment decisions based on medical consultation	27 (56.3%)	40 (78.4%)	37 (74.0%)	0.041	0.259	0.207

Data are presented as *n* (%). *p* denotes the raw probability value; *q* denotes the Benjamini–Hochberg FDR-adjusted value across 79 testable hypotheses. *q* < 0.05 is shown in bold. Different superscript letters indicate significant Bonferroni-corrected pairwise differences (α = 0.0167); groups sharing a letter did not differ. *V*: Cramér’s *V*. Complete response distributions for all items are provided in File S2.

**Table 3:** Multivariable logistic regression analysis of factors associated with denosumab recognition and routine medication-history questioning.

Outcome/Predictor	aOR (95% CI)	*p*
**Denosumab selected as an MRONJ-associated agent**		
General dentist vs. student	0.39 (0.11–1.43)	0.157
Specialist vs. student	0.47 (0.11–1.90)	0.287
Age 25–34 vs. <25	0.64 (0.19–2.21)	0.482
Age ≥ 35 vs. <25	0.60 (0.15–2.42)	0.472
Male vs. female	1.00 (0.48–2.09)	0.990
**Routine history-taking for antiresorptive/antiangiogenic drug use**		
General dentist vs. student	0.29 (0.08–1.03)	0.056
Specialist vs. student	0.39 (0.10–1.54)	0.179
Age 25–34 vs. <25	1.33 (0.39–4.55)	0.652
Age ≥ 35 vs. <25	0.78 (0.19–3.18)	0.730
Male vs. female	1.15 (0.57–2.35)	0.697

aOR: adjusted odds ratio; CI: confidence interval. Models were mutually adjusted for professional status, age group, and sex. Reference categories were student, age < 25 years, and female. For the denosumab model, the overall effect of professional status was LR *p* = 0.366; for the routine history-taking model, LR *p* = 0.140. *N* = 149 in both models.

## Data Availability

The data supporting the findings of this study are available from the corresponding author upon reasonable request. The data are not publicly available because of ethical and participant-privacy considerations.

## Supplementary Materials

The following supporting information can be downloaded at https://www.mdpi.com/article/10.3390/healthcare14172819/s1: File S1: Questionnaire on Knowledge and Awareness of Medication-Related Osteonecrosis of the Jaw (MRONJ); Table S1: Item-level test–retest reliability and agreement statistics [13,14]; File S2: Detailed Statistical Analysis Results; Table S2: Descriptive distribution of key MRONJ knowledge and clinical-approach items across the eight individual dental specialties.

## Abbreviations

MRONJ, medication-related osteonecrosis of the jaw; AAOMS, American Association of Oral and Maxillofacial Surgeons; FDR, false discovery rate; aOR, adjusted odds ratio; CI, confidence interval; CTX, C-terminal telopeptide; CVI, Content Validity Index; ICC, intraclass correlation coefficient; RANKL, receptor activator of nuclear factor-κB ligand; VEGF, vascular endothelial growth factor.
