# Supplementary material for: Knowledge and Self-Reported Clinical Approaches Regarding Medication-Related Osteonecrosis of the Jaw Among Final-Year Dental Students, General Dentists, and Specialist Dentists: An Exploratory Cross-Sectional Study
Source: Healthcare (Basel). 2026 Sep 2;14(17):2819. doi: 10.3390/healthcare14172819 (PMC13565098; doi:10.3390/healthcare14172819)
Supplement: Supplementary file 1 [file healthcare-14-02819-s001.zip › MRONJ_SupplementaryTableS2-edited.pdf]

Supplementary Table S2. Descriptive distribution of key MRONJ knowledge and clinical-approach items across the eight individual dental specialties.

Data are presented as n (%) within each specialty. Owing to the small number of participants in each specialty (n = 4–8), only descriptive results are provided; no between-specialty significance tests were conducted. The "All specialists" column shows the pooled specialist results reported in Table 2 of the main text.

Item	Oral and Maxillofacial Radiology (n = 6)	Oral and Maxillofacial Surgery (n = 8)	Endodontics (n = 8)	Prosthodontics (n = 6)	Restorative Dentistry (n = 6)	Periodontology (n = 6)	Pediatric Dentistry (n = 6)	Orthodontics (n = 4)	All specialists (n = 50)
Bone metastases of solid tumours [a]	4 (66.7%)	8 (100.0%)	7 (87.5%)	4 (66.7%)	6 (100.0%)	5 (83.3%)	6 (100.0%)	2 (50.0%)	**42 (84.0%)**
Multiple myeloma [a]	5 (83.3%)	6 (75.0%)	2 (25.0%)	1 (16.7%)	2 (33.3%)	3 (50.0%)	3 (50.0%)	1 (25.0%)	**23 (46.0%)**
Alendronate [b]	4 (66.7%)	8 (100.0%)	6 (75.0%)	1 (16.7%)	1 (16.7%)	5 (83.3%)	4 (66.7%)	0 (0.0%)	**29 (58.0%)**
Pamidronate [b]	4 (66.7%)	7 (87.5%)	3 (37.5%)	3 (50.0%)	3 (50.0%)	4 (66.7%)	4 (66.7%)	1 (25.0%)	**29 (58.0%)**
Zoledronate [b]	6 (100.0%)	7 (87.5%)	7 (87.5%)	3 (50.0%)	5 (83.3%)	5 (83.3%)	4 (66.7%)	3 (75.0%)	**40 (80.0%)**
Denosumab [b]	5 (83.3%)	7 (87.5%)	3 (37.5%)	3 (50.0%)	1 (16.7%)	3 (50.0%)	3 (50.0%)	2 (50.0%)	**27 (54.0%)**
Antiresorptive/antiangiogenic medication history [c]	2 (33.3%)	7 (87.5%)	5 (62.5%)	6 (100.0%)	0 (0.0%)	2 (33.3%)	4 (66.7%)	0 (0.0%)	**26 (52.0%)**

MRONJ, medication-related osteonecrosis of the jaw. [a] Identified as an indication for bisphosphonate therapy. [b] Identified as a medication associated with osteonecrosis of the jaw. [c] Reported routinely asking about antiresorptive or antiangiogenic medication use as part of the medical history. Percentages were calculated within each specialty; specialties are listed in the same order as in Table 1.
