# Supplementary material for: Knowledge and Self-Reported Clinical Approaches Regarding Medication-Related Osteonecrosis of the Jaw Among Final-Year Dental Students, General Dentists, and Specialist Dentists: An Exploratory Cross-Sectional Study
Source: Healthcare (Basel). 2026 Sep 2;14(17):2819. doi: 10.3390/healthcare14172819 (PMC13565098; doi:10.3390/healthcare14172819)
Supplement: Supplementary file 1 [file healthcare-14-02819-s001.zip › MRONJ_Supplementary_Material_S2(1)(1)-edited.pdf]

Supplementary Material S2: Detailed Statistical Analysis Results.

Statistical methods: Frequencies and percentages were used as descriptive statistics. The distributions of variables were assessed using the Kolmogorov–Smirnov and Shapiro–Wilk tests. Categorical independent data were analyzed using the chi-square test, and Fisher's exact test was used when the chi-square assumptions were not met. Analyses were performed using SPSS version 27.0.

Table 1

		n	%
Age	<25	55	36.9%
	25-34	68	45.6%
	35-50	23	15.4%
	>50	3	2.0%
Gender	Female	51	34.2%
	Male	98	65.8%
Professional Status	Student	48	32.2%
	General Dentist	51	34.2%
	Specialist Dentist	50	33.6%
Specialty			
(-)		99	66.4%
Oral and Maxillofacial Radiology		6	4.0%
Oral and Maxillofacial Surgery		8	5.4%
Endodontics		8	5.4%
Prosthodontics		6	4.0%
Restorative Dentistry		6	4.0%
Periodontology		6	4.0%
Pediatric Dentistry		6	4.0%
Orthodontics		4	2.7%
Clinical Experience	Final-year student	48	32.2%
	1–3 years	29	19.5%
	3–5 years	18	12.1%
	5–10 years	28	18.8%
	>10 years	26	17.4%

Table 2

		n	%
General Awareness of Dentistry and MRONJ			
Have You Ever Heard of Medication-Related Osteonecrosis of the Jaw (MRONJ)?			
This Is the First Time I Have Heard of It		4	2.7%
I Have Heard of It but Do Not Know What It Is		3	2.0%
I Have Some Knowledge		79	53.0%
I Have Sufficient Knowledge		63	42.3%
What Are the Diagnostic Criteria for MRONJ According to the American Association of Oral and Maxillofacial Surgeons?			
Current or Previous Treatment With Antiresorptive or Antiangiogenic Agents		93	62.4%
Exposed Bone or an Intraoral/Extraoral Fistula in the Maxillofacial Region Persisting for More Than 8 Weeks		113	75.8%
No History of Radiation Therapy to the Jaws		36	24.2%
No History of Metastatic Disease Involving the Jaws		21	14.1%
I Am Not Sure		19	12.8%
Are You Familiar With Antiresorptive and Antiangiogenic Medications?			
This Is the First Time I Have Heard of It		10	6.7%
I Have Heard of It but Do Not Know What It Is		21	14.1%
I Have Some Knowledge		94	63.1%
I Have Sufficient Knowledge		24	16.1%
Where Have You Previously Heard About Antiresorptive and Antiangiogenic Medications?			
I Have Never Heard of Them Before		9	6.0%
University Education		132	88.6%
Media		11	7.4%
Scientific Journals		25	16.8%
Scientific Meetings/Conferences		20	13.4%
How Long Do Bisphosphonates Remain Pharmacologically Active in the Body After Treatment Has Ended?	Up to 6 Months	23	15.4%
	Up to 1 Year	16	10.7%
	Up to 10 Years	95	63.8%
	I Do Not Know	15	10.1%

Table 3

		n	%
Therapeutic Use of Antiresorptive and Antiangiogenic Drugs			
Which Medication(s) May Lead to MRONJ With Long-Term Use?	Antiresorptive Drugs	55	36.9%
	Bisphosphonates	139	93.3%
	Antidiabetic Drugs	3	2.0%
	Antihypertensive Drugs	1	0.7%
	Corticosteroids	21	14.1%
	Anticoagulant/Antiplatelet Drugs	7	4.7%
	RANKL Inhibitors	61	40.9%
	I Am Not Sure	4	2.7%
For Which Conditions Are Bisphosphonates Used?	Cancer-Related Hypercalcemia	85	57.0%
	Bone Metastases From Solid Tumors	97	65.1%
	Paget's Disease	67	45.0%
	Multiple Myeloma	54	36.2%
	Fibrous Dysplasia	33	22.1%
	Osteoporosis	115	77.2%
	Multiple Sclerosis	20	13.4%
	Osteogenesis Imperfecta	23	15.4%
Which Active Pharmaceutical Ingredients May Cause Osteonecrosis?	Acebutolol	19	12.8%
	Sunitinib	19	12.8%
	Alendronate	61	40.9%
	Pamidronate	64	43.0%
	Zoledronate	89	59.7%
	Denosumab	91	61.1%
	Bromocriptine	4	2.7%
	Bevacizumab	36	24.2%
To Which Class of Medications Does Denosumab Belong?	Bisphosphonate	43	28.9%
	RANKL Inhibitor	28	18.8%
	Antiangiogenic Agent	16	10.7%
	I Am Not Sure	62	41.6%
Which Factors Increase the Potency of Bisphosphonates?	Intravenous Formulation	94	63.1%
	Number of Radical Groups in the Chemical Structure	38	25.5%
	Nitrogen Content in the Chemical Structure	27	18.1%
	Tablet (Oral) Formulation	2	1.3%
	I Do Not Know	44	29.5%
Compared With Intravenous Administration, Does Routine Oral Use of Bisphosphonates Carry a Higher Risk of Developing MRONJ?	Yes	56	37.6%
	No	93	62.4%
Which of the Following Are Considered Risk Factors for MRONJ?	Poor Oral Hygiene	128	85.9%
	Periodontal Disease	125	83.9%
	Tobacco Use	104	69.8%
	Corticosteroid Therapy	100	67.1%
	Hypertension	42	28.2%
	Hyperlipidemia	36	24.2%

	Alcohol Consumption	81	54.4%

Table 4

		n	%
**Dental Management of Patients With MRONJ**			
Which Findings Should Be Carefully Evaluated During the Clinical and Radiographic Examination of Patients With MRONJ?	Halitosis	68	45.6%
	Mucosal Ulceration/Inflammation	107	71.8%
	Tooth Mobility	67	45.0%
	Exposed Bone	142	95.3%
	Thickening of the Lamina Dura	43	28.9%
	Widening of the Periodontal Ligament Space	68	45.6%
Does Your Routine Medical History Include the Question: 'Are You Currently Using or Have You Previously Used Antiresorptive or Antiangiogenic Medications?'	Yes	82	55.0%
	No	67	45.0%
A 47-Year-Old Female Patient Who Has Been Taking Oral Alendronic Acid for Osteoporosis for One Year and Has No Other Systemic Disease Presents for Tooth Extraction. Would You Perform the Surgical Procedure?			
Yes		46	30.9%
No		103	69.1%
If You Decide to Perform the Surgical Procedure, Would You Implement Any Additional Measures?	Yes	140	94.0%
	No	9	6.0%
Additional Measures			
Preoperative Antibiotic Prophylaxis		88	59.1%
Postoperative Antibiotic Prophylaxis		61	40.9%
Consultation With the Physician Who Prescribed the Medication		132	88.6%
Assessment of the Serum CTX Level Before Surgery		91	61.1%
Other		7	4.7%
In Your Opinion, What Serum CTX Level Should Be Considered Acceptable to Minimize the Risk of Osteonecrosis During Surgical Procedures?	<100 pg/ml	46	30.9%
	100-125 pg/ml	40	26.8%
	126-149 pg/ml	12	8.1%
	>149 pg/ml	51	34.2%
How Would You Approach a Patient Receiving Antiresorptive or Antiangiogenic Therapy?			
I Would Not Provide Treatment		17	11.4%
I Would Provide Only Nonsurgical Treatment		36	24.2%
I Would Perform Minor Surgical Procedures (e.g., Tooth Extraction)		18	12.1%
I Would Perform Surgical Procedures (e.g., Implant Placement)		3	2.0%
I Would Provide All Necessary Treatments Based on Medical Consultation		104	69.8%
Before Tooth Extraction or Implant Placement, for How Long Would You Recommend Discontinuing Anti-Osteoporotic Medication?	≤2 Months	17	11.4%
	3–5 Months	24	16.1%
	6 Months	108	72.5%
How Would You Plan Dental Treatment Before Initiating Intravenous Bisphosphonate Therapy?			

Endodontic Treatment for Teeth With Apical Lesions	84	56.4%
Endodontic Treatment for Teeth With Deep Carious Lesions	89	59.7%
Extraction of Teeth With Deep Periodontal Pockets	86	57.7%
Extraction of Retained Root Fragments	100	67.1%
Monitoring of Impacted Teeth	81	54.4%
Extraction of Teeth Likely to Cause Pericoronitis	91	61.1%
I Do Not Know	23	15.4%

Age was significantly higher in the general dentist and specialist dentist groups than in the student group ($p<0.05$). Age did not differ significantly between the general dentist and specialist dentist groups ($p>0.05$). Gender distribution did not differ significantly among the student, general dentist, and specialist dentist groups ($p>0.05$) (Table 5).

Clinical experience was significantly greater in the general dentist and specialist dentist groups than in the student group ($p<0.05$). Clinical experience did not differ significantly between the general dentist and specialist dentist groups ($p>0.05$) (Table 5).

Table 5

		[1] Student (n=48)		[2] General Dentist (n=51)		[3] Specialist Dentist (n=50)		p	
		n	%	n	%	n	%		
Age									
<25		45	93.8%	8	15.7%	2	4.0%	***0.001***	X^2
25-34		3	6.3%	29	56.9%	36	72.0%		
35-50		0	0.0%	12	23.5%	11	22.0%		
>50		0[23]	0.0%	2	3.9%	1	2.0%		
Gender	Female	17	35.4%	21	41.2%	13	26.0%	0.269	X^2
	Male	31	64.6%	30	58.8%	37	74.0%		
Clinical Experience									
Final-year student		48	100%	0	0.0%	0	0.0%	***0.000***	X^2
1–3 years		0	0.0%	19	37.3%	10	20.0%		
3–5 years		0	0.0%	8	15.7%	10	20.0%		
5–10 years		0	0.0%	13	25.5%	15	30.0%		
>10 years		0[23]	0.0%	11	21.6%	15	30.0%		

χ^2 test (Fisher's exact test where appropriate)

[2]Different from the general dentist group, $p<0.05$; [3]Different from the specialist dentist group, $p<0.05$

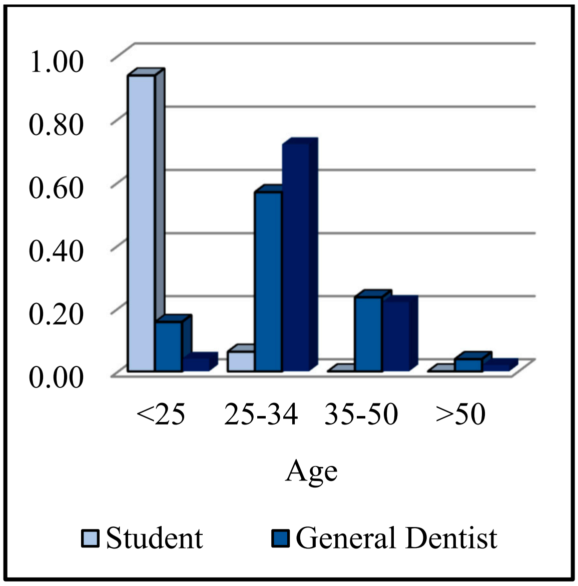

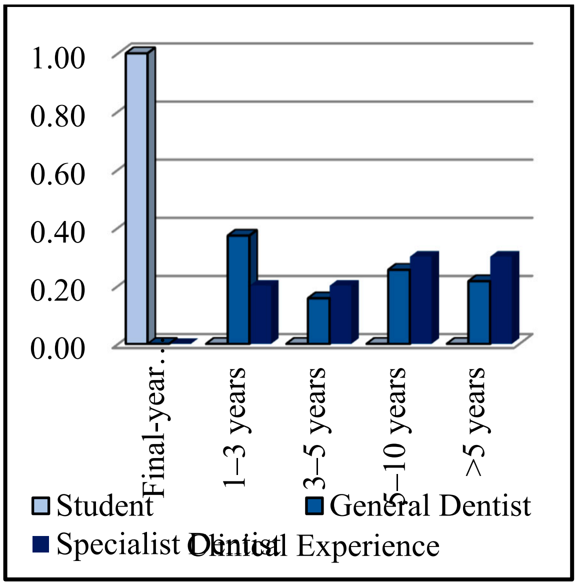

The proportion of participants familiar with medication-related osteonecrosis of the jaw (MRONJ) did not differ significantly among the student, general dentist, and specialist dentist groups (p>0.05) (Table 6).

The proportion selecting 'current or previous treatment with antiresorptive or antiangiogenic agents' as an AAOMS diagnostic criterion for MRONJ was significantly higher in the student group than in the general dentist and specialist dentist groups (p<0.05) (Table 6).

The proportion selecting 'current or previous treatment with antiresorptive or antiangiogenic agents' did not differ significantly between the general dentist and specialist dentist groups (p>0.05) (Table 6).

The proportion selecting 'exposed bone or an intraoral/extraoral fistula in the maxillofacial region persisting for more than 8 weeks' as an AAOMS diagnostic criterion was significantly higher in the specialist dentist group than in the general dentist group (p<0.05) (Table 6).

The proportion selecting this diagnostic criterion did not differ significantly between the student group and the general dentist or specialist dentist groups (p>0.05) (Table 6).

The proportion selecting 'No History of Radiation Therapy to the Jaws' in response to 'What Are the Diagnostic Criteria for MRONJ According to the American Association of Oral and Maxillofacial Surgeons?' did not differ significantly among the student, general dentist, and specialist dentist groups (p>0.05) (Table 6).

The proportion selecting 'No History of Metastatic Disease Involving the Jaws' in response to 'What Are the Diagnostic Criteria for MRONJ According to the American Association of Oral and Maxillofacial Surgeons?' did not differ significantly among the student, general dentist, and specialist dentist groups (p>0.05) (Table 6).

The proportion selecting ‘I am not sure’ for the AAOMS diagnostic criteria did not differ significantly among the student, general dentist, and specialist dentist groups (p>0.05) (Table 6).

Table 6

General Awareness of Dentistry and MRONJ	[1] Student (n=48)		[2] General Dentist (n=51)		[3] Specialist Dentist (n=50)		p
	n	%	n	%	n	%	
Have You Ever Heard of Medication-Related Osteonecrosis of the Jaw (MRONJ)?							
This Is the First Time I Have Heard of It	0	0.0%	2	3.9%	2	4.0%	
I Have Heard of It but Do Not Know What It Is	1	2.1%	1	2.0%	1	2.0%	0.582 χ^2
I Have Some Knowledge	26	54.2%	30	58.8%	23	46.0%	
I Have Sufficient Knowledge	21	43.8%	18	35.3%	24	48.0%	
What Are the Diagnostic Criteria for MRONJ According to the American Association of Oral and Maxillofacial Surgeons?							
Current or Previous Treatment With Antiresorptive or Antiangiogenic Agents	37	77.1%	27[1]	52.9%	29[1]	58.0%	***0.034*** χ^2
Exposed Bone or an Intraoral/Extraoral Fistula in the Maxillofacial Region Persisting for More Than 8 Weeks	37	77.1%	33[3]	64.7%	43	86.0%	***0.043*** χ^2
No History of Radiation Therapy to the Jaws	14	29.2%	8	15.7%	14	28.0%	0.217 χ^2
No History of Metastatic Disease Involving the Jaws	8	16.7%	4	7.8%	9	18.0%	0.281 χ^2
I Am Not Sure	3	6.3%	10	19.6%	6	12.0%	0.135 χ^2

χ^2 test (Fisher’s exact test where appropriate)

[1] Different from the student group, p<0.05; [3] Different from the specialist dentist group, p<0.05

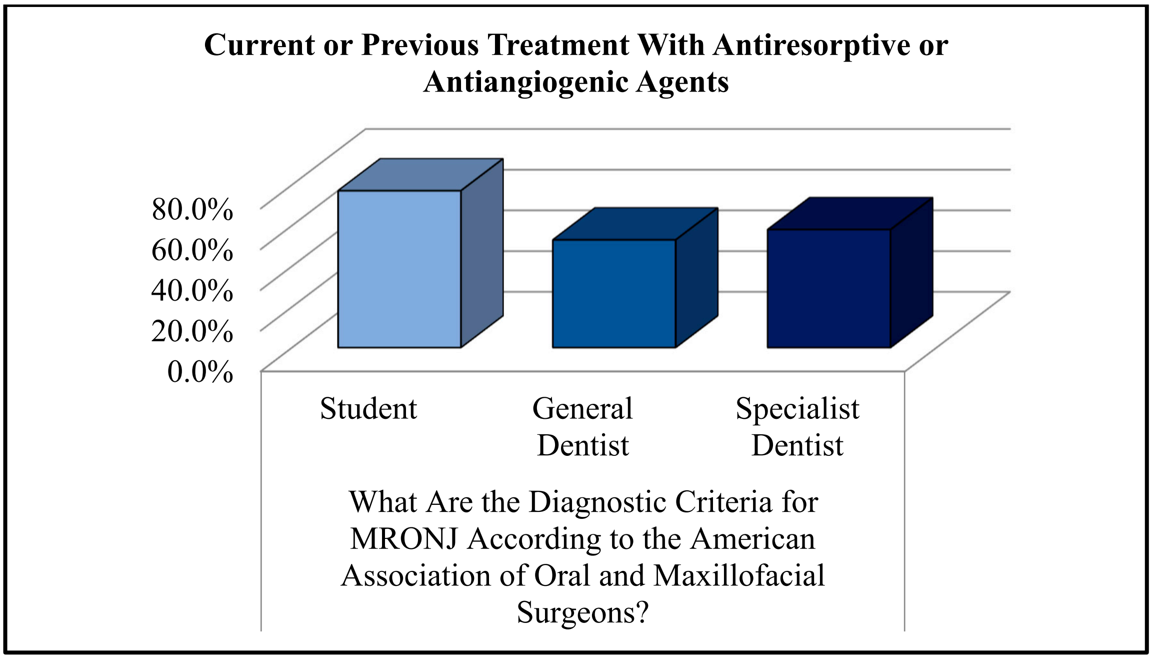

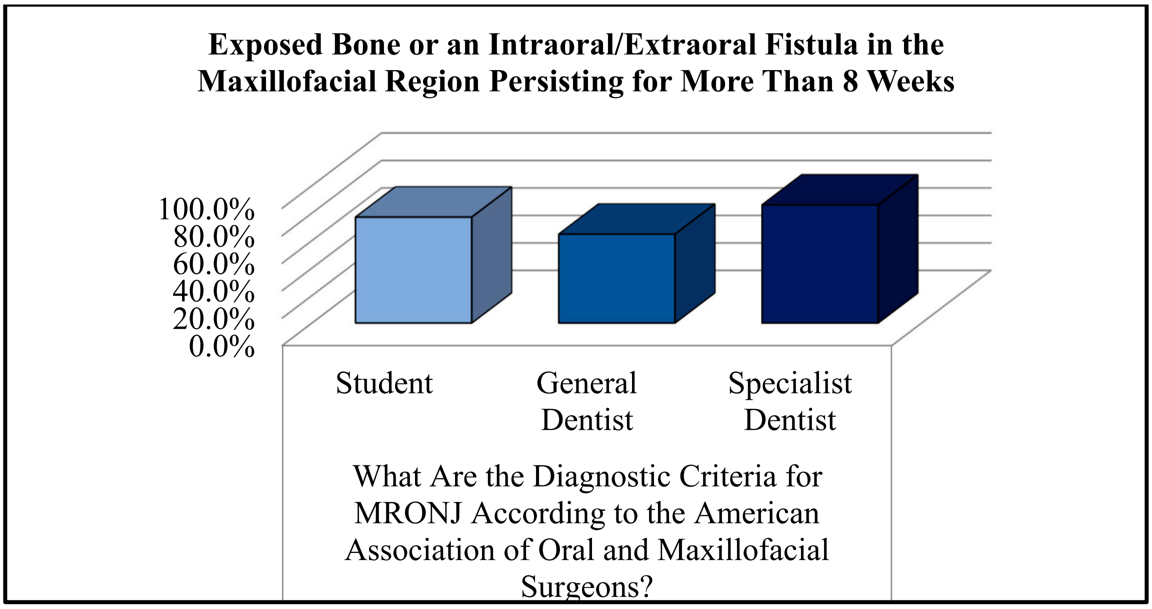

Self-reported knowledge of antiresorptive and antiangiogenic drugs did not differ significantly among the student, general dentist, and specialist dentist groups ($p>0.05$) (Table 7).

The proportion selecting 'I Have Never Heard of Them Before' in response to 'Where Have You Previously Heard About Antiresorptive and Antiangiogenic Medications?' did not differ significantly among the student, general dentist, and specialist dentist groups ($p>0.05$) (Table 7).

The proportion reporting university education as a source of information about antiresorptive and antiangiogenic drugs was significantly higher in the student group than in the general dentist and specialist dentist groups ($p<0.05$). No significant difference was observed between the general dentist and specialist dentist groups ($p>0.05$) (Table 7).

The proportion selecting ‘Media’ in response to ‘Where Have You Previously Heard About Antiresorptive and Antiangiogenic Medications?’ did not differ significantly among the student, general dentist, and specialist dentist groups (p>0.05) (Table 7).

The proportion reporting scientific journals as a source of information was significantly higher in the specialist dentist group than in the student and general dentist groups (p<0.05). No significant difference was observed between the student and general dentist groups (p>0.05) (Table 7).

The proportion selecting ‘Scientific Meetings/Conferences’ in response to ‘Where Have You Previously Heard About Antiresorptive and Antiangiogenic Medications?’ did not differ significantly among the student, general dentist, and specialist dentist groups (p>0.05) (Table 7).

The distribution of responses regarding how long bisphosphonates remain active in the body after treatment did not differ significantly among the groups (p>0.05) (Table 7).

Table 7

General Awareness of Dentistry and MRONJ	[1] Student (n=48)		[2] General Dentist (n=51)		[3] Specialist Dentist (n=50)		p
	n	%	n	%	n	%	
Are You Familiar With Antiresorptive and Antiangiogenic Medications?							
This Is the First Time I Have Heard of It	1	2.1%	4	7.8%	5	10.0%	
I Have Heard of It but Do Not Know What It Is	5	10.4%	11	21.6%	5	10.0%	0.152 χ^2
I Have Some Knowledge	33	68.8%	32	62.7%	29	58.0%	
I Have Sufficient Knowledge	9	18.8%	4	7.8%	11	22.0%	
Where Have You Previously Heard About Antiresorptive and Antiangiogenic Medications?							
I Have Never Heard of Them Before	0	0.0%	4	7.8%	5	10.0%	0.093 χ^2
University Education	48	100%	41[1]	80.4%	43[1]	86.0%	***0.007*** χ^2
Media	4	8.3%	4	7.8%	3	6.0%	0.896 χ^2
Scientific Journals	5[3]	10.4%	5[3]	9.8%	15	30.0%	***0.009*** χ^2
Scientific Meetings/Conferences	4	8.3%	7	13.7%	9	18.0%	0.372 χ^2
**How Long Do Bisphosphonates Remain Pharmacologically Active in the Body After Treatment Has Ended?**							
Up to 6 Months	7	14.6%	9	17.6%	7	14.0%	
Up to 1 Year	4	8.3%	7	13.7%	5	10.0%	0.815 χ^2
Up to 10 Years	33	68.8%	28	54.9%	34	68.0%	
I Do Not Know	4	8.3%	7	13.7%	4	8.0%	

χ^2 test (Fisher’s exact test where appropriate)

[1] Different from the student group, p<0.05; [3] Different from the specialist dentist group, p<0.05.

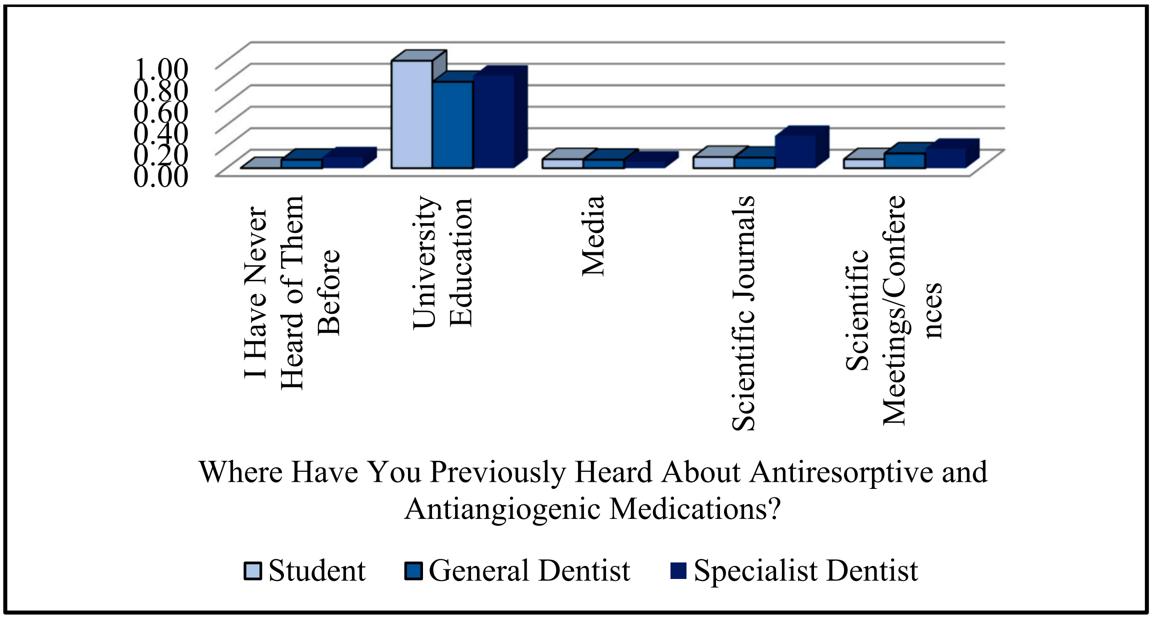

The proportion selecting antiresorptive drugs as medications that may lead to MRONJ with long-term use was significantly higher in the student and specialist dentist groups than in the general dentist group ($p<0.05$). No significant difference was observed between the student and specialist dentist groups ($p>0.05$) (Table 8).

The proportion selecting 'Bisphosphonates' in response to 'Which Medication(s) May Lead to MRONJ With Long-Term Use?' did not differ significantly among the student, general dentist, and specialist dentist groups ($p>0.05$) (Table 8).

The proportion selecting 'Antidiabetic Drugs' in response to 'Which Medication(s) May Lead to MRONJ With Long-Term Use?' did not differ significantly among the student, general dentist, and specialist dentist groups ($p>0.05$) (Table 8).

The proportion selecting 'Antihypertensive Drugs' in response to 'Which Medication(s) May Lead to MRONJ With Long-Term Use?' did not differ significantly among the student, general dentist, and specialist dentist groups ($p>0.05$) (Table 8).

The proportion selecting 'Corticosteroids' in response to 'Which Medication(s) May Lead to MRONJ With Long-Term Use?' did not differ significantly among the student, general dentist, and specialist dentist groups ($p>0.05$) (Table 8).

The proportion selecting 'Anticoagulant/Antiplatelet Drugs' in response to 'Which Medication(s) May Lead to MRONJ With Long-Term Use?' did not differ significantly among the student, general dentist, and specialist dentist groups ($p>0.05$) (Table 8).

The proportion selecting ‘RANKL Inhibitors’ in response to ‘Which Medication(s) May Lead to MRONJ With Long-Term Use?’ did not differ significantly among the student, general dentist, and specialist dentist groups (p>0.05) (Table 8).

The proportion selecting ‘I Am Not Sure’ in response to ‘Which Medication(s) May Lead to MRONJ With Long-Term Use?’ did not differ significantly among the student, general dentist, and specialist dentist groups (p>0.05) (Table 8).

Table 8

Therapeutic Use of Antiresorptive and Antiangiogenic Drugs	[1] Student (n=48)		[2] General Dentist (n=51)		[3] Specialist Dentist (n=50)		p	
	n	%	n	%	n	%		
Which Medication(s) May Lead to MRONJ With Long-Term Use?								
Antiresorptive Drugs	21	43.8%	10[13]	19.6%	24	48.0%	***0.006***	X^2
Bisphosphonates	47	97.9%	45	88.2%	47	94.0%	0.152	X^2
Antidiabetic Drugs	1	2.1%	2	3.9%	0	0.0%	p>0.05	X^2
Antihypertensive Drugs	0	0.0%	1	2.0%	0	0.0%	p>0.05	X^2
Corticosteroids	5	10.4%	8	15.7%	8	16.0%	0.673	X^2
Anticoagulant/Antiplatelet Drugs	2	4.2%	3	5.9%	2	4.0%	0.885	X^2
RANKL Inhibitors	19	39.6%	21	41.2%	21	42.0%	0.970	X^2
I Am Not Sure	0	0.0%	2	3.9%	2	4.0%	p>0.05	X^2

χ^2 test (Fisher’s exact test where appropriate)

[1] Different from the student group, p<0.05; [3] Different from the specialist dentist group, p<0.05.

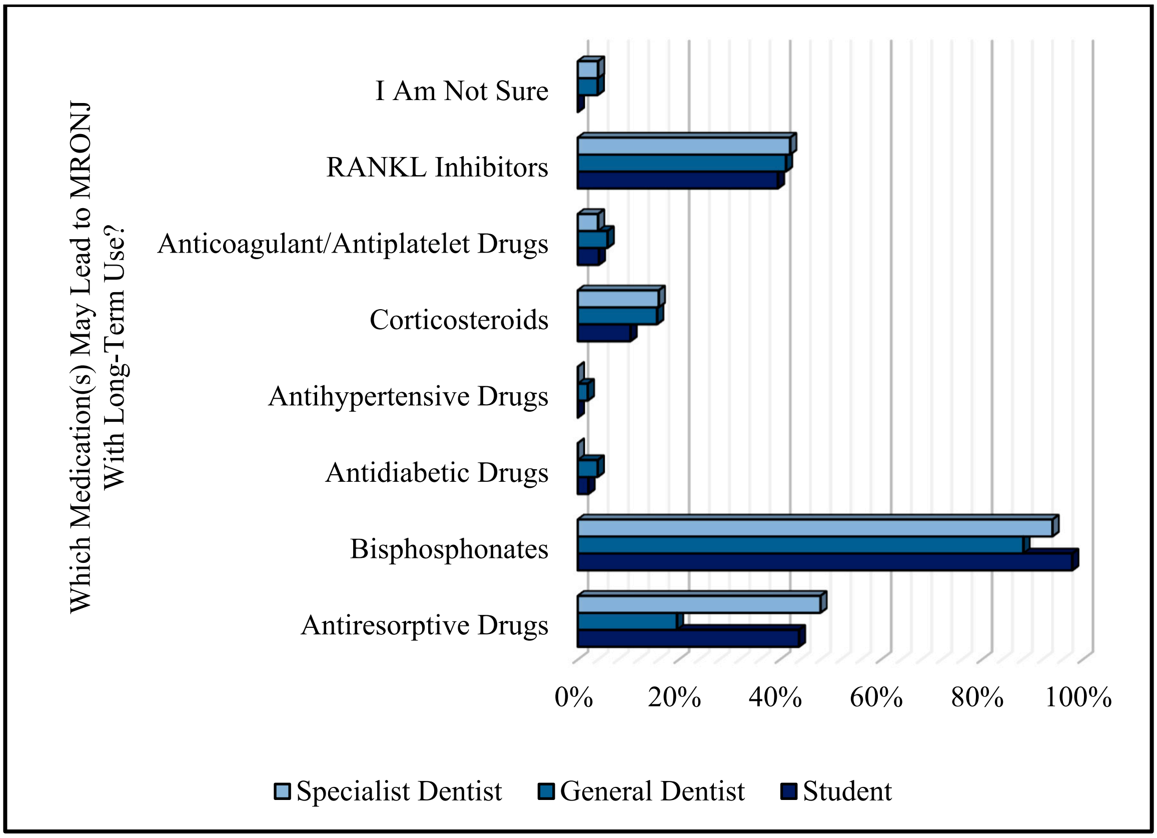

The proportion selecting 'Cancer-Related Hypercalcemia' in response to 'For Which Conditions Are Bisphosphonates Used?' did not differ significantly among the student, general dentist, and specialist dentist groups ($p>0.05$) (Table 9).

The proportion selecting bone metastases from solid tumors as an indication for bisphosphonates was significantly higher in the specialist dentist group than in the student and general dentist groups ($p<0.05$) (Table 9).

No significant difference was observed between the student and general dentist groups for this response ($p>0.05$) (Table 9).

The proportion selecting Paget's disease as an indication for bisphosphonates did not differ significantly among the student, general dentist, and specialist dentist groups ($p>0.05$) (Table 9).

The proportion selecting multiple myeloma as an indication for bisphosphonates was significantly higher in the general dentist and specialist dentist groups than in the student group ($p<0.05$) (Table 9).

No significant difference was observed between the general dentist and specialist dentist groups for this response ($p>0.05$) (Table 9).

The proportion selecting fibrous dysplasia as an indication for bisphosphonates did not differ significantly among the student, general dentist, and specialist dentist groups (p>0.05) (Table 9).

The proportion selecting osteoporosis as an indication for bisphosphonates did not differ significantly among the student, general dentist, and specialist dentist groups (p>0.05) (Table 9).

The proportion selecting multiple sclerosis as an indication for bisphosphonates did not differ significantly among the student, general dentist, and specialist dentist groups (p>0.05) (Table 9).

The proportion selecting osteogenesis imperfecta as an indication for bisphosphonates did not differ significantly among the student, general dentist, and specialist dentist groups (p>0.05) (Table 9).

Table 9

Therapeutic Use of Antiresorptive and Antiangiogenic Drugs	[1] Student (n=48)		[2] General Dentist (n=51)		[3] Specialist Dentist (n=50)		p	
	n	%	n	%	n	%		
For Which Conditions Are Bisphosphonates Used?								
Cancer-Related Hypercalcemia	25	52.1%	28	54.9%	32	64.0%	0.457	X^2
Bone Metastases From Solid Tumors	27[3]	56.3%	28[3]	54.9%	42	84.0%	***0.003***	X^2
Paget's Disease	24	50.0%	17	33.3%	26	52.0%	0.118	X^2
Multiple Myeloma	8[2,3]	16.7%	23	45.1%	23	46.0%	***0.003***	X^2
Fibrous Dysplasia	16	33.3%	9	17.6%	8	16.0%	0.075	X^2
Osteoporosis	37	77.1%	36	70.6%	42	84.0%	0.275	X^2
Multiple Sclerosis	3	6.3%	8	15.7%	9	18.0%	0.197	X^2
Osteogenesis Imperfecta	10	20.8%	7	13.7%	6	12.0%	0.441	X^2

χ^2 test; [2] Different from the general dentist group, p<0.05; [3]Different from the specialist dentist group, p<0.05

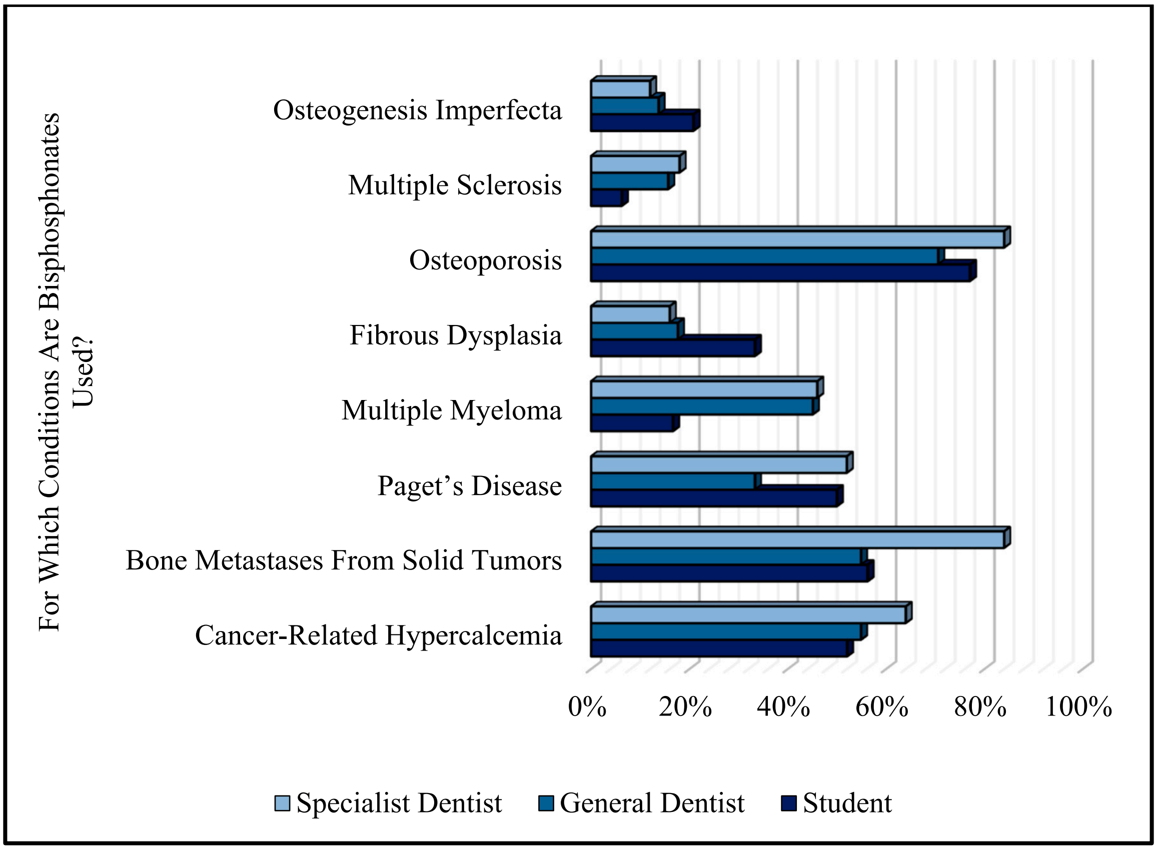

The proportion selecting ‘Acebutolol’ in response to ‘Which Active Pharmaceutical Ingredients May Cause Osteonecrosis?’ did not differ significantly among the student, general dentist, and specialist dentist groups ($p>0.05$) (Table 10).

The proportion selecting ‘Sunitinib’ in response to ‘Which Active Pharmaceutical Ingredients May Cause Osteonecrosis?’ did not differ significantly among the student, general dentist, and specialist dentist groups ($p>0.05$) (Table 10).

The proportion identifying alendronate as an agent that may cause osteonecrosis was significantly higher in the general dentist and specialist dentist groups than in the student group ($p<0.05$). No significant difference was observed between the general dentist and specialist dentist groups ($p>0.05$) (Table 10).

The proportion identifying pamidronate as an agent that may cause osteonecrosis was significantly higher in the general dentist and specialist dentist groups than in the student group ($p<0.05$). No significant difference was observed between the general dentist and specialist dentist groups ($p>0.05$) (Table 10).

The proportion identifying zoledronate as an agent that may cause osteonecrosis was significantly higher in the general dentist and specialist dentist groups than in the student group ($p<0.05$). No significant difference was observed between the general dentist and specialist dentist groups ($p>0.05$) (Table 10).

The proportion identifying denosumab as an agent that may cause osteonecrosis was significantly higher in the student group than in the general dentist and specialist dentist groups ($p<0.05$). No significant difference was observed between the general dentist and specialist dentist groups ($p>0.05$) (Table 10).

The proportion selecting ‘Bromocriptine’ in response to ‘Which Active Pharmaceutical Ingredients May Cause Osteonecrosis?’ did not differ significantly among the student, general dentist, and specialist dentist groups ($p>0.05$) (Table 10).

The proportion selecting ‘Bevacizumab’ in response to ‘Which Active Pharmaceutical Ingredients May Cause Osteonecrosis?’ did not differ significantly among the student, general dentist, and specialist dentist groups ($p>0.05$) (Table 10).

The distribution of responses regarding the pharmacological class of denosumab did not differ significantly among the student, general dentist, and specialist dentist groups ($p>0.05$) (Table 10).

Table 10

Therapeutic Use of Antiresorptive and Antiangiogenic Drugs	[1] Student (n=48)		[2] General Dentist (n=51)		[3] Specialist Dentist (n=50)		p	
	n	%	n	%	n	%		
Which Active Pharmaceutical Ingredients May Cause Osteonecrosis?								
Acebutolol	7	14.6%	8	15.7%	4	8.0%	0.460	X^2
Sunitinib	7	14.6%	7	13.7%	5	10.0%	0.768	X^2
Alendronate	9[2,3]	18.8%	23	45.1%	29	58.0%	***0.000***	X^2
Pamidronate	11[2,3]	22.9%	24	47.1%	29	58.0%	***0.002***	X^2
Zoledronate	17[2,3]	35.4%	32	62.7%	40	80.0%	***0.000***	X^2
Denosumab	38	79.2%	26[1]	51.0%	27[1]	54.0%	***0.007***	X^2
Bromocriptine	2	4.2%	1	2.0%	1	2.0%	p>0.05	X^2
Bevacizumab	14	29.2%	9	17.6%	13	26.0%	0.381	X^2
To Which Class of Medications Does Denosumab Belong?								
Bisphosphonate	21	43.8%	11	21.6%	11	22.0%	0.116	X^2
RANKL Inhibitor	8	16.7%	11	21.6%	9	18.0%		
Antiangiogenic Agent	6	12.5%	6	11.8%	4	8.0%		
I Am Not Sure	13	27.1%	23	45.1%	26	52.0%		

χ^2 test (Fisher’s exact test where appropriate); [1]Different from the student group, $p<0.05$

[2] Different from the general dentist group, $p<0.05$; [3]Different from the specialist dentist group, $p<0.05$.

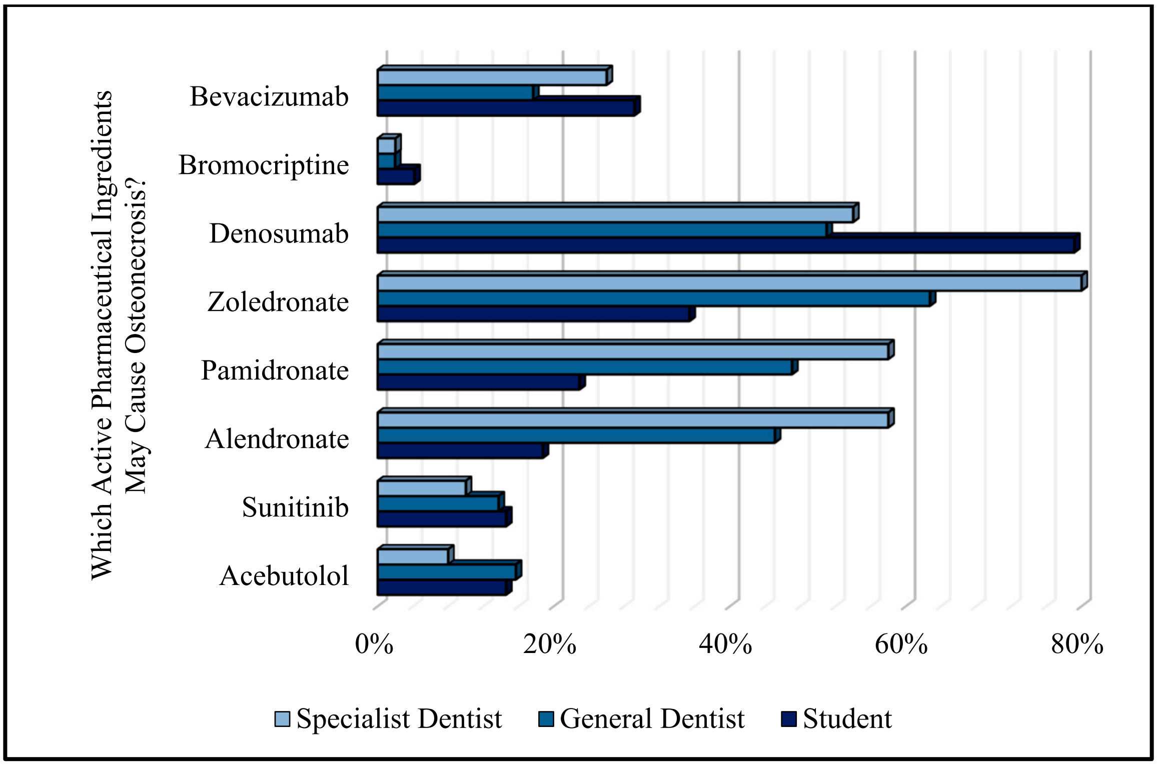

The proportion selecting 'Intravenous Formulation' in response to 'Which Factors Increase the Potency of Bisphosphonates?' did not differ significantly among the student, general dentist, and specialist dentist groups (p>0.05) (Table 11).

The proportion selecting 'Number of Radical Groups in the Chemical Structure' in response to 'Which Factors Increase the Potency of Bisphosphonates?' did not differ significantly among the student, general dentist, and specialist dentist groups (p>0.05) (Table 11).

The proportion selecting 'Nitrogen Content in the Chemical Structure' in response to 'Which Factors Increase the Potency of Bisphosphonates?' did not differ significantly among the student, general dentist, and specialist dentist groups (p>0.05) (Table 11).

The proportion selecting 'Tablet (Oral) Formulation' in response to 'Which Factors Increase the Potency of Bisphosphonates?' did not differ significantly among the student, general dentist, and specialist dentist groups (p>0.05) (Table 11).

The proportion selecting 'I Do Not Know' in response to 'Which Factors Increase the Potency of Bisphosphonates?' did not differ significantly among the student, general dentist, and specialist dentist groups (p>0.05) (Table 11).

The distribution of responses regarding whether routine oral bisphosphonate use carries a higher risk of MRONJ than intravenous administration did not differ significantly among the groups ($p>0.05$) (Table 11).

Table 11

Therapeutic Use of Antiresorptive and Antiangiogenic Drugs	Student (n=48)		General Dentist (n=51)		Specialist Dentist (n=50)		p
	n	%	n	%	n	%	
Which Factors Increase the Potency of Bisphosphonates?							
Intravenous Formulation	30	62.5%	28	54.9%	36	72.0%	0.204 X^2
Number of Radical Groups in the Chemical Structure	11	22.9%	17	33.3%	10	20.0%	0.271 X^2
Nitrogen Content in the Chemical Structure	4	8.3%	11	21.6%	12	24.0%	0.097 X^2
Tablet (Oral) Formulation	1	2.1%	0	0.0%	1	2.0%	$p>0.05$ X^2
I Do Not Know	13	27.1%	17	33.3%	14	28.0%	0.760 X^2
Compared With Intravenous Administration, Does Routine Oral Use of Bisphosphonates Carry a Higher Risk of Developing MRONJ?							
Yes	19	39.6%	22	43.1%	15	30.0%	0.372 X^2
No	29	60.4%	29	56.9%	35	70.0%	

χ^2 test (Fisher's exact test where appropriate)

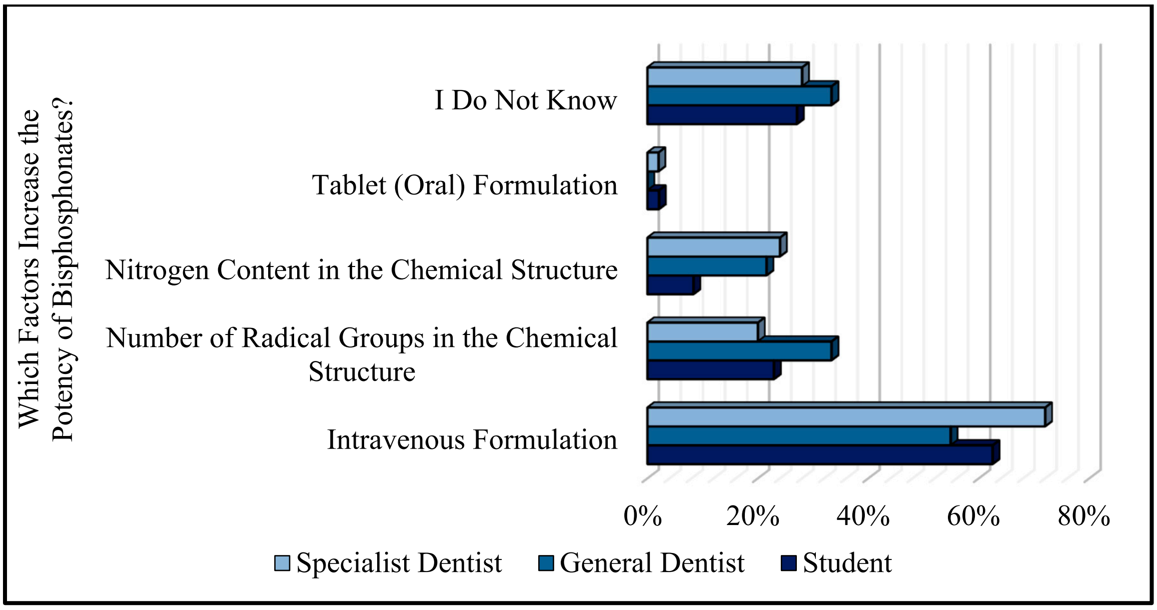

The proportion selecting 'Poor Oral Hygiene' in response to 'Which of the Following Are Considered Risk Factors for MRONJ?' did not differ significantly among the student, general dentist, and specialist dentist groups ($p>0.05$) (Table 12).

The proportion selecting ‘Periodontal Disease’ in response to ‘Which of the Following Are Considered Risk Factors for MRONJ?’ did not differ significantly among the student, general dentist, and specialist dentist groups (p>0.05) (Table 12).

The proportion selecting ‘Tobacco Use’ in response to ‘Which of the Following Are Considered Risk Factors for MRONJ?’ did not differ significantly among the student, general dentist, and specialist dentist groups (p>0.05) (Table 12).

The proportion selecting ‘Corticosteroid Therapy’ in response to ‘Which of the Following Are Considered Risk Factors for MRONJ?’ did not differ significantly among the student, general dentist, and specialist dentist groups (p>0.05) (Table 12).

The proportion selecting ‘Hypertension’ in response to ‘Which of the Following Are Considered Risk Factors for MRONJ?’ did not differ significantly among the student, general dentist, and specialist dentist groups (p>0.05) (Table 12).

The proportion selecting ‘Hyperlipidemia’ in response to ‘Which of the Following Are Considered Risk Factors for MRONJ?’ did not differ significantly among the student, general dentist, and specialist dentist groups (p>0.05) (Table 12).

The proportion selecting ‘Alcohol Consumption’ in response to ‘Which of the Following Are Considered Risk Factors for MRONJ?’ did not differ significantly among the student, general dentist, and specialist dentist groups (p>0.05) (Table 12).

Table 12

Therapeutic Use of Antiresorptive and Antiangiogenic Drugs	Student (n=48)		General Dentist (n=51)		Specialist Dentist (n=50)		p	
	n	%	n	%	n	%		
Which of the Following Are Considered Risk Factors for MRONJ?								
Poor Oral Hygiene	43	89.6%	40	78.4%	45	90.0%	0.167	X^2
Periodontal Disease	43	89.6%	39	76.5%	43	86.0%	0.183	X^2
Tobacco Use	34	70.8%	33	64.7%	37	74.0%	0.586	X^2
Corticosteroid Therapy	34	70.8%	31	60.8%	35	70.0%	0.493	X^2
Hypertension	17	35.4%	13	25.5%	12	24.0%	0.395	X^2
Hyperlipidemia	15	31.3%	10	19.6%	11	22.0%	0.364	X^2
Alcohol Consumption	30	62.5%	24	47.1%	27	54.0%	0.304	X^2

χ^2 test

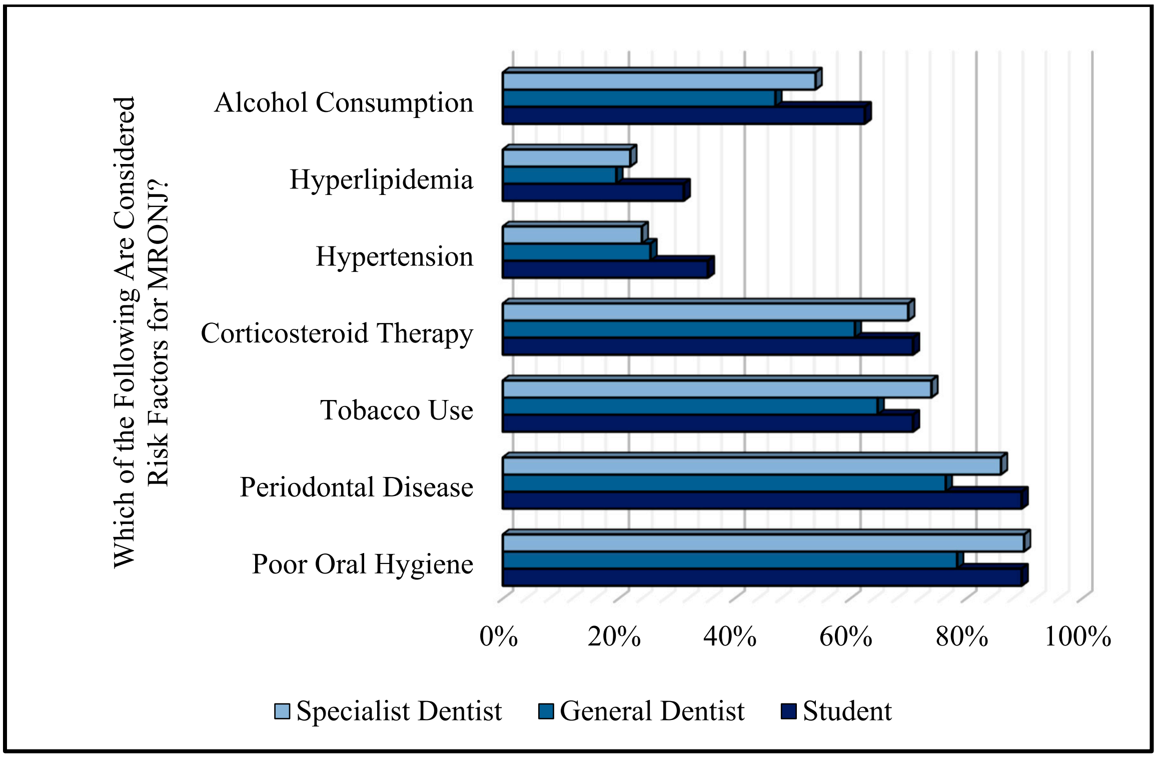

The proportion selecting 'Halitosis' in response to 'Which Findings Should Be Carefully Evaluated During the Clinical and Radiographic Examination of Patients With MRONJ?' did not differ significantly among the student, general dentist, and specialist dentist groups (p>0.05) (Table 13).

The proportion selecting 'Mucosal Ulceration/Inflammation' in response to 'Which Findings Should Be Carefully Evaluated During the Clinical and Radiographic Examination of Patients With MRONJ?' did not differ significantly among the student, general dentist, and specialist dentist groups (p>0.05) (Table 13).

The proportion selecting 'Tooth Mobility' in response to 'Which Findings Should Be Carefully Evaluated During the Clinical and Radiographic Examination of Patients With MRONJ?' did not differ significantly among the student, general dentist, and specialist dentist groups (p>0.05) (Table 13).

The proportion selecting 'Exposed Bone' in response to 'Which Findings Should Be Carefully Evaluated During the Clinical and Radiographic Examination of Patients With MRONJ?' did not differ significantly among the student, general dentist, and specialist dentist groups (p>0.05) (Table 13).

The proportion selecting 'Widening of the Periodontal Ligament Space' in response to 'Which Findings Should Be Carefully Evaluated During the Clinical and Radiographic Examination of Patients With MRONJ?' did not differ significantly among the student, general dentist, and specialist dentist groups (p>0.05) (Table 13).

The proportion reporting that antiresorptive or antiangiogenic drug use was routinely questioned during medical history taking was significantly higher in the student group than in the general dentist group ($p<0.05$). No significant difference was observed between the specialist dentist group and the other two groups ($p>0.05$) (Table 13).

Table 13

Dental Management of Patients With MRONJ	[1] Student (n=48)		[2] General Dentist (n=51)		[3] Specialist Dentist (n=50)		p	
	n	%	n	%	n	%		
Which Findings Should Be Carefully Evaluated During the Clinical and Radiographic Examination of Patients With MRONJ?								
Halitosis	25	52.1%	22	43.1%	21	42.0%	0.549	X^2
Mucosal Ulceration/Inflammation	33	68.8%	33	64.7%	41	82.0%	0.131	X^2
Tooth Mobility	20	41.7%	24	47.1%	23	46.0%	0.851	X^2
Exposed Bone	47	97.9%	47	92.2%	48	96.0%	0.384	X^2
Thickening of the Lamina Dura	15	31.3%	14	27.5%	14	28.0%	0.904	X^2
Widening of the Periodontal Ligament Space	24	50.0%	24	47.1%	20	40.0%	0.591	X^2
Does Your Routine Medical History Include the Question: 'Are You Currently Using or Have You Previously Used Antiresorptive or Antiangiogenic Medications?'								
Yes	34	70.8%	22[1]	43.1%	26	52.0%	***0.019***	X^2
No	14	29.2%	29	56.9%	24	48.0%		

χ^2 test (Fisher's exact test where appropriate); [1]Different from the student group, $p<0.05$

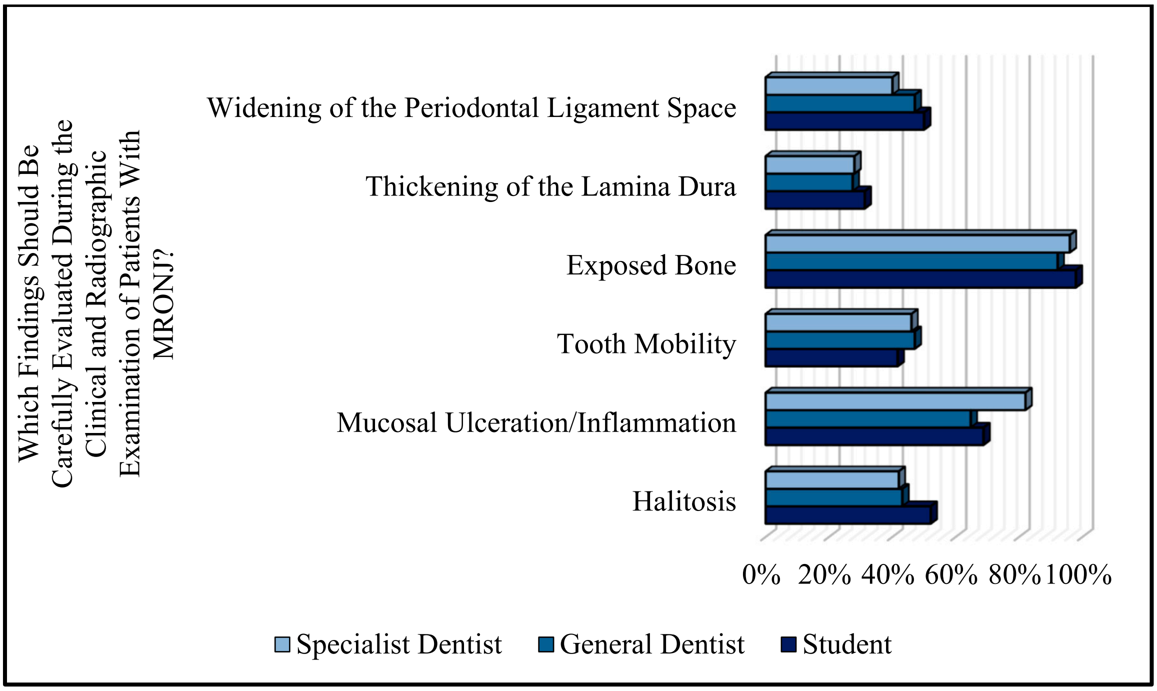

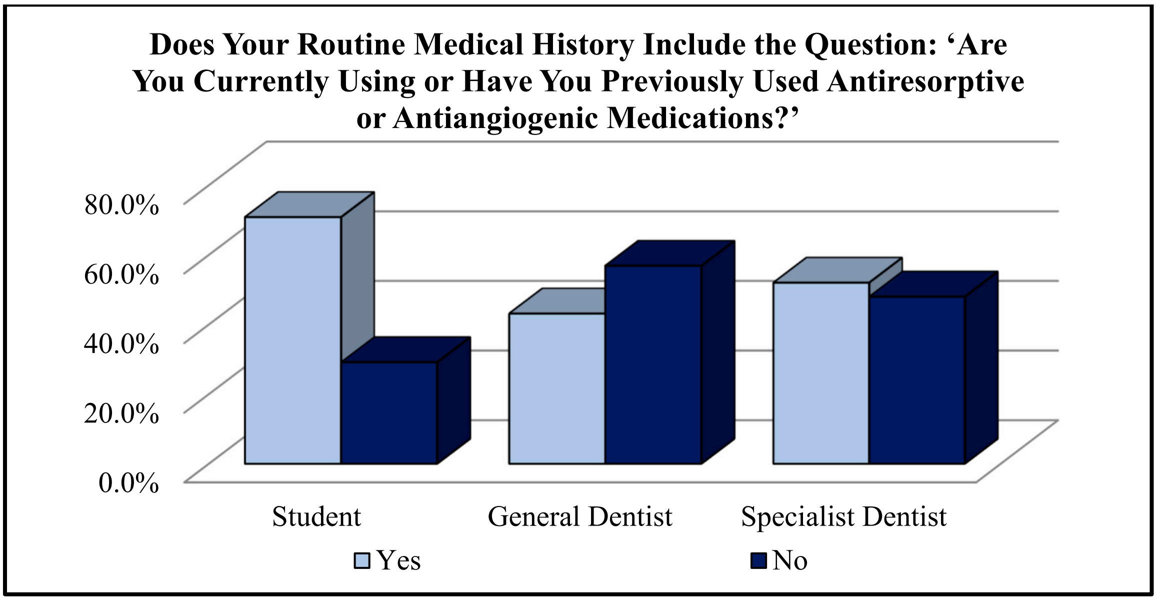

The proportion that would perform a surgical procedure in the presented clinical scenario did not differ significantly among the student, general dentist, and specialist dentist groups (p>0.05) (Table 14).

The proportion that would implement additional measures when performing the surgical procedure did not differ significantly among the groups (p>0.05) (Table 14).

The proportions selecting preoperative antibiotic prophylaxis, postoperative antibiotic prophylaxis, consultation with the prescribing physician, preoperative serum CTX assessment, or other additional measures did not differ significantly among the groups (p>0.05) (Table 14).

The distribution of responses regarding the serum CTX threshold considered acceptable for minimizing osteonecrosis risk during surgery did not differ significantly among the groups (p>0.05) (Table 14).

Table 14

Dental Management of Patients With MRONJ	Student (n=48)		General Dentist (n=51)		Specialist Dentist (n=50)		p	
	n	%	n	%	n	%		
A 47-Year-Old Female Patient Who Has Been Taking Oral Alendronic Acid for Osteoporosis for One Year and Has No Other Systemic Disease Presents for Tooth Extraction. Would You Perform the Surgical Procedure?								
Yes	15	31.3%	16	31.4%	15	30.0%	0.987	X^2
No	33	68.8%	35	68.6%	35	70.0%		
If You Decide to Perform the Surgical Procedure, Would You Implement Any Additional Measures?								
Yes	45	93.8%	47	92.2%	48	96.0%	0.718	X^2
No	3	6.3%	4	7.8%	2	4.0%		
Additional Measures								
Preoperative Antibiotic Prophylaxis	29	60.4%	28	54.9%	31	62.0%	0.748	X^2
Postoperative Antibiotic Prophylaxis	16	33.3%	20	39.2%	25	50.0%	0.234	X^2
Consultation With the Physician Who Prescribed the Medication	43	89.6%	42	82.4%	47	94.0%	0.178	X^2
Assessment of the Serum CTX Level Before Surgery	26	54.2%	30	58.8%	35	70.0%	0.253	X^2
Other	1	2.1%	5	9.8%	1	2.0%	0.105	X^2
In Your Opinion, What Serum CTX Level Should Be Considered Acceptable to Minimize the Risk of Osteonecrosis During Surgical Procedures?								
<100 pg/ml	12	25.0%	14	27.5%	20	40.0%	0.102	
100-125 pg/ml	12	25.0%	19	37.3%	9	18.0%		
126-149 pg/ml	7	14.6%	1	2.0%	4	8.0%		
>149 pg/ml	17	35.4%	17	33.3%	17	34.0%		

χ^2 test (Fisher's exact test where appropriate)

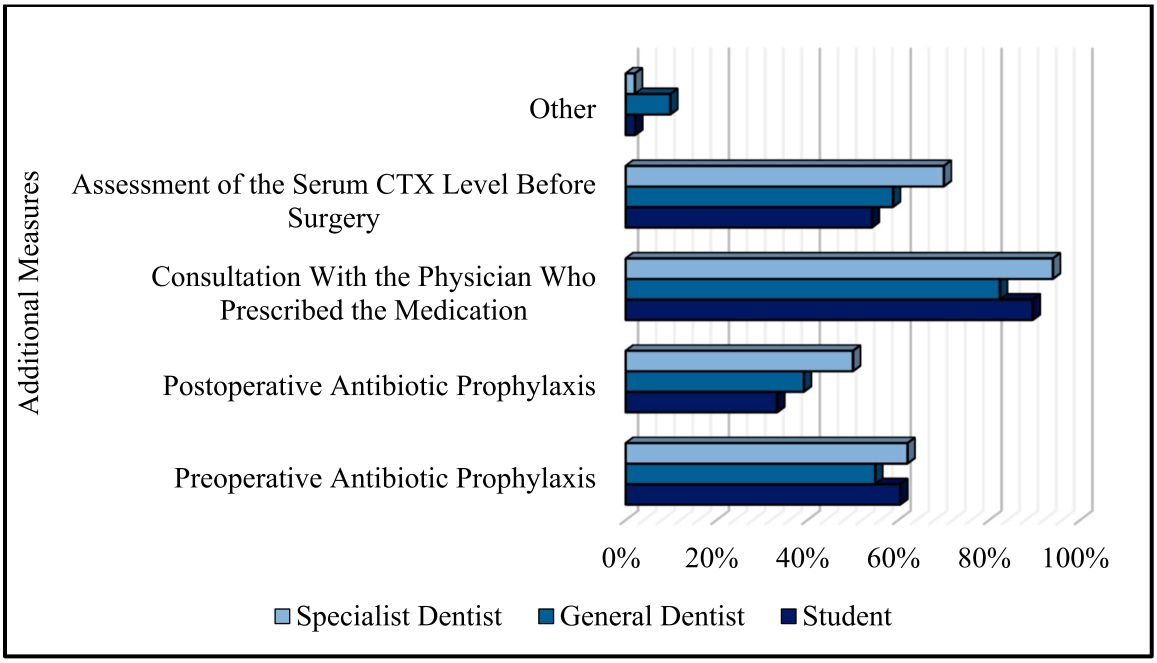

The proportion selecting 'I Would Not Provide Treatment' in response to 'How Would You Approach a Patient Receiving Antiresorptive or Antiangiogenic Therapy?' did not differ significantly among the student, general dentist, and specialist dentist groups ($p>0.05$) (Table 15).

The proportion selecting 'I Would Provide Only Nonsurgical Treatment' in response to 'How Would You Approach a Patient Receiving Antiresorptive or Antiangiogenic Therapy?' did not differ significantly among the student, general dentist, and specialist dentist groups ($p>0.05$) (Table 15).

The proportion selecting 'I Would Perform Minor Surgical Procedures' in response to 'How Would You Approach a Patient Receiving Antiresorptive or Antiangiogenic Therapy?' did not differ significantly among the student, general dentist, and specialist dentist groups ($p>0.05$) (Table 15).

The proportion selecting 'I Would Perform Surgical Procedures' in response to 'How Would You Approach a Patient Receiving Antiresorptive or Antiangiogenic Therapy?' did not differ significantly among the student, general dentist, and specialist dentist groups ($p>0.05$) (Table 15).

The proportion selecting 'I would provide all necessary treatments based on medical consultation' was significantly higher in the general dentist group than in the student group ($p<0.05$). No significant difference was observed between the specialist dentist group and the other two groups ($p>0.05$) (Table 15).

The distribution of recommended discontinuation periods for anti-osteoporotic medication before tooth extraction or implant placement did not differ significantly among the groups (p>0.05) (Table 15).

Table 15

Dental Management of Patients With MRONJ	[1] Student (n=48)		[2] General Dentist (n=51)		[3] Specialist Dentist (n=50)		p
	n	%	n	%	n	%	
How Would You Approach a Patient Receiving Antiresorptive or Antiangiogenic Therapy?							
I Would Not Provide Treatment	3	6.3%	4	7.8%	10	20.0%	0.062 $^{X^2}$
I Would Provide Only Nonsurgical Treatment	16	33.3%	9	17.6%	11	22.0%	0.173 $^{X^2}$
I Would Perform Minor Surgical Procedures	6	12.5%	7	13.7%	5	10.0%	0.843 $^{X^2}$
I Would Perform Surgical Procedures	0	0.0%	1	2.0%	2	4.0%	p>0.05 $^{X^2}$
I Would Provide All Necessary Treatments Based on Medical Consultation	27[2]	56.3%	40	78.4%	37	74.0%	***0.041*** $^{X^2}$
Before Tooth Extraction or Implant Placement, for How Long Would You Recommend Discontinuing Anti-Osteoporotic Medication?							
≤2 Months	10	20.8%	5	9.8%	2	4.0%	
3–5 Months	6	12.5%	9	17.6%	9	18.0%	0.123
6 Months	32	66.7%	37	72.5%	39	78.0%	

χ^2 test (Fisher's exact test where appropriate); [2] Different from the general dentist group, p<0.05

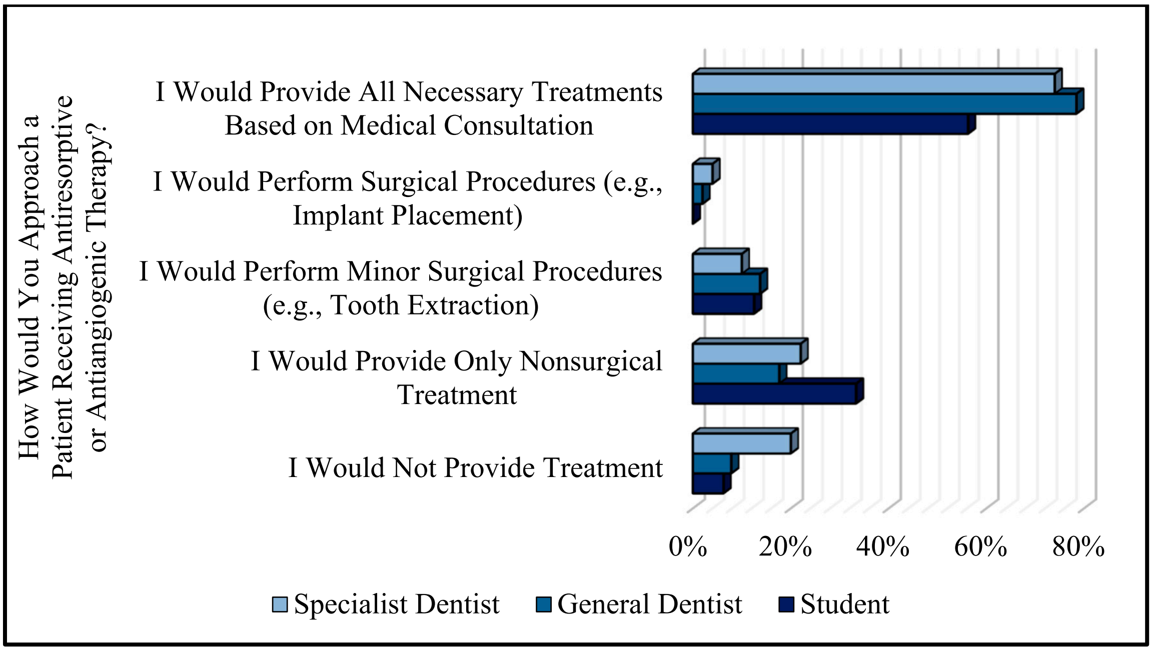

The proportion selecting endodontic treatment for teeth with apical lesions when planning dental treatment before intravenous bisphosphonate therapy did not differ significantly among the groups (p>0.05) (Table 16).

The proportion selecting endodontic treatment for teeth with deep carious lesions did not differ significantly among the groups (p>0.05) (Table 16).

The proportion selecting extraction of teeth with deep periodontal pockets did not differ significantly among the groups (p>0.05) (Table 16).

The proportion selecting extraction of retained root fragments did not differ significantly among the groups (p>0.05) (Table 16).

The proportion selecting monitoring of impacted teeth did not differ significantly among the groups (p>0.05) (Table 16).

The proportions selecting extraction of teeth likely to cause pericoronitis and 'I do not know' when planning dental treatment before intravenous bisphosphonate therapy did not differ significantly among the groups (p>0.05) (Table 16).

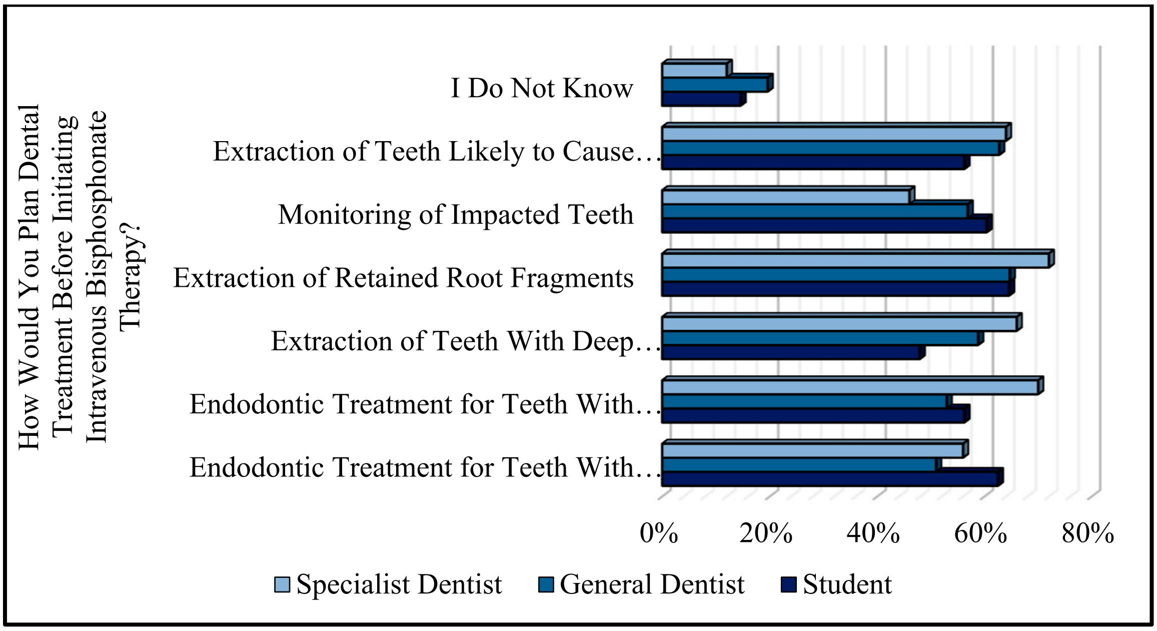

Table 16

Dental Management of Patients With MRONJ	Student (n=48)		General Dentist (n=51)		Specialist Dentist (n=50)		p
	n	%	n	%	n	%	
How Would You Plan Dental Treatment Before Initiating Intravenous Bisphosphonate Therapy?							
Endodontic Treatment for Teeth With Apical Lesions	30	62.5%	26	51.0%	28	56.0%	0.512 $^{X^2}$

Endodontic Treatment for Teeth With Deep Carious Lesions	27	56.3%	27	52.9%	35	70.0%	0.182	X^2
Extraction of Teeth With Deep Periodontal Pockets	23	47.9%	30	58.8%	33	66.0%	0.190	X^2
Extraction of Retained Root Fragments	31	64.6%	33	64.7%	36	72.0%	0.666	X^2
Monitoring of Impacted Teeth	29	60.4%	29	56.9%	23	46.0%	0.325	X^2
Extraction of Teeth Likely to Cause Pericoronitis	27	56.3%	32	62.7%	32	64.0%	0.701	X^2
I Do Not Know	7	14.6%	10	19.6%	6	12.0%	0.560	X^2

χ^2 test
