# Supplementary material for: Knowledge and Self-Reported Clinical Approaches Regarding Medication-Related Osteonecrosis of the Jaw Among Final-Year Dental Students, General Dentists, and Specialist Dentists: An Exploratory Cross-Sectional Study
Source: Healthcare (Basel). 2026 Sep 2;14(17):2819. doi: 10.3390/healthcare14172819 (PMC13565098; doi:10.3390/healthcare14172819)
Supplement: Supplementary file 1 [file healthcare-14-02819-s001.zip › MRONJ_Supplementary_Table_S1(2)-edited.pdf]

Supplementary Table S1. Item-level test–retest reliability and agreement statistics.

Question	Item format	Exact agreement (%)	Cohen's κ	κ interpretation	Spearman's ρ	ρ interpretation	p value
**Q5**	Single-response	93.3	0.874	Almost perfect	0.886	Strong	<0.001
**Q6**	Multiple-response	83.3	0.785	Substantial	0.907	Very strong	<0.001
**Q7**	Single-response	93.3	0.816	Almost perfect	0.920	Very strong	<0.001
**Q8**	Multiple-response	83.3	0.675	Substantial	0.677	Moderate	<0.001
**Q9**	Single-response	86.7	0.705	Substantial	0.809	Strong	<0.001
**Q10**	Multiple-response	80.0	0.711	Substantial	0.715	Strong	<0.001
**Q11**	Multiple-response	56.7	0.549	Moderate	0.873	Strong	<0.001
**Q12**	Multiple-response	46.7	0.408	Moderate	0.731	Strong	<0.001
**Q13**	Single-response	90.0	0.853	Almost perfect	0.829	Strong	<0.001
**Q14**	Multiple-response	80.0	0.752	Substantial	0.803	Strong	<0.001
**Q15**	Single-response	83.3	0.667	Substantial	0.668	Moderate	<0.001
**Q16**	Multiple-response	73.3	0.705	Substantial	0.776	Strong	<0.001
**Q17**	Multiple-response	63.3	0.583	Moderate	0.828	Strong	<0.001
**Q18**	Single-response	90.0	0.796	Substantial	0.813	Strong	<0.001
**Q19**	Single-response	90.0	0.780	Substantial	0.783	Strong	<0.001
**Q20**	Single-response	96.7	0.870	Almost perfect	0.877	Strong	<0.001
**Q21**	Multiple-response	60.0	0.510	Moderate	0.677	Moderate	<0.001
**Q22**	Single-response	86.7	0.805	Almost perfect	0.758	Strong	<0.001
**Q23**	Multiple-response	70.0	0.581	Moderate	0.648	Moderate	<0.001
**Q24**	Single-response	90.0	0.709	Substantial	0.836	Strong	<0.001
**Q25**	Multiple-response	60.0	0.549	Moderate	0.793	Strong	<0.001

Abbreviations and analysis note. Q, question; κ, Cohen's kappa; ρ, Spearman's rank correlation coefficient. Exact agreement denotes identical response categories for single-response items and identical sets of selected options for multiple-response items across the two administrations. For the supplementary Spearman analysis, single-response items were analyzed using the coded response category and multiple-response items using the number of selected options.

Interpretation thresholds. Cohen's κ: ≤0.00, poor; >0.00–0.20, slight; >0.20–0.40, fair; >0.40–0.60, moderate; >0.60–0.80, substantial; and >0.80–1.00, almost perfect agreement. Absolute Spearman's ρ: 0.00–0.09, negligible; 0.10–0.39, weak; 0.40–0.69, moderate; 0.70–0.89, strong; and 0.90–1.00, very strong correlation.

References. 1. Landis JR, Koch GG. The measurement of observer agreement for categorical data. Biometrics. 1977;33(1):159–174. 2. Schober P, Boer C, Schwarte LA. Correlation coefficients: appropriate use and interpretation. Anesth Analg. 2018;126(5):1763–1768.
