# Supplementary material for: Knowledge and Self-Reported Clinical Approaches Regarding Medication-Related Osteonecrosis of the Jaw Among Final-Year Dental Students, General Dentists, and Specialist Dentists: An Exploratory Cross-Sectional Study
Source: Healthcare (Basel). 2026 Sep 2;14(17):2819. doi: 10.3390/healthcare14172819 (PMC13565098; doi:10.3390/healthcare14172819)
Supplement: Supplementary file 1 [file healthcare-14-02819-s001.zip › Supplementary_Material_S1(2)-edited.pdf]

Supplementary Material S1

Questionnaire on Knowledge and Awareness of Medication-Related Osteonecrosis of the Jaw (MRONJ)

This study aims to investigate the level of knowledge and awareness regarding medication-related osteonecrosis of the jaw (MRONJ) among dentists. The study is conducted by Assistant Professor Çiğdem Şeker and Research Assistant İsmail Çapar from the Department of Oral and Maxillofacial Radiology, Faculty of Dentistry, Zonguldak Bülent Ecevit University. Ethical approval has been obtained from the Non-Interventional Clinical Research Ethics Committee of Zonguldak Bülent Ecevit University.

We sincerely appreciate your participation and valuable contribution to this research.

*** Indicates a required question.**

No personally identifiable information will be collected in this questionnaire investigating dentists' knowledge and awareness of MRONJ. All responses will be kept strictly confidential in accordance with the applicable legislation concerning the protection of personal data and will be used solely for scientific research purposes by the investigators. By reading and approving this form, you indicate your voluntary consent to participate in the study. However, you have the right to decline participation or withdraw from the study at any time without any consequences.

I have read and understood the information above and voluntarily agree to participate in this study.

- ☐ Yes
- ☐ No

A. Demographic Information

1. Age

- ☐ <25 years
- ☐ 25–34 years
- ☐ 35–50 years
- ☐ >50 years

2. Gender

- ☐ Male

- ☐ Female

3. Educational/Professional Status

- ☐ Final-year dental student
- ☐ General dentist
- ☐ Specialist dentist

If you are a specialist dentist, please indicate your specialty:

- ☐ Oral and Maxillofacial Radiology
- ☐ Oral and Maxillofacial Surgery
- ☐ Endodontics
- ☐ Prosthodontics
- ☐ Restorative Dentistry
- ☐ Periodontology
- ☐ Pediatric Dentistry
- ☐ Orthodontics

4. Years of Clinical Experience

- ☐ Final-year dental student
- ☐ 1–3 years
- ☐ 3–5 years
- ☐ 5–10 years
- ☐ More than 10 years

B. Questions on General Awareness of Dentistry and MRONJ

5. Have you ever heard of medication-related osteonecrosis of the jaw (MRONJ)?

- ☐ This is the first time I have heard of it.
- ☐ I have heard of it but do not know what it is.
- ☐ I have some knowledge about it.
- ☐ I have sufficient knowledge about it.

6. According to the American Association of Oral and Maxillofacial Surgeons (AAOMS), what are the diagnostic criteria for MRONJ? (Select all that apply.)

- ☐ Current or previous treatment with antiresorptive or antiangiogenic agents
- ☐ Exposed bone or an intraoral/extraoral fistula in the maxillofacial region persisting for more than 8 weeks

- ☐ No history of radiation therapy to the jaws
- ☐ No history of metastatic disease involving the jaws
- ☐ I am not sure.

7. Are you familiar with antiresorptive and antiangiogenic medications?

- ☐ This is the first time I have heard of them.
- ☐ I have heard of them but do not know what they are.
- ☐ I have some knowledge about them.
- ☐ I have sufficient knowledge about them.

8. Where have you previously heard about antiresorptive and antiangiogenic medications? (Select all that apply.)

- ☐ I have never heard of them before.
- ☐ University education
- ☐ Media
- ☐ Scientific journals
- ☐ Scientific meetings/conferences

9. How long do bisphosphonates remain pharmacologically active in the body after treatment has ended?

- ☐ Up to 6 months
- ☐ Up to 1 year
- ☐ Up to 10 years
- ☐ I do not know

C. Questions on the Therapeutic Use of Antiresorptive and Antiangiogenic Drugs

10. Which medication(s) may lead to MRONJ with long-term use? (Select all that apply.)

- ☐ Antiresorptive drugs
- ☐ Bisphosphonates
- ☐ Antidiabetic drugs
- ☐ Antihypertensive drugs
- ☐ Corticosteroids
- ☐ Anticoagulant/antiplatelet drugs
- ☐ RANKL inhibitors
- ☐ I am not sure

11. For which conditions are bisphosphonates used? (Select all that apply.)

- ☐ Cancer-related hypercalcemia
- ☐ Bone metastases from solid tumors
- ☐ Paget’s disease
- ☐ Multiple myeloma
- ☐ Fibrous dysplasia
- ☐ Osteoporosis
- ☐ Multiple sclerosis
- ☐ Osteogenesis imperfecta

12. Which active pharmaceutical ingredients may cause osteonecrosis? (Select all that apply.)

- ☐ Acebutolol
- ☐ Sunitinib
- ☐ Alendronate
- ☐ Pamidronate
- ☐ Zoledronate
- ☐ Denosumab
- ☐ Bromocriptine
- ☐ Bevacizumab

13. Denosumab belongs to which class of medications?

- ☐ Bisphosphonate
- ☐ RANKL inhibitor
- ☐ Antiangiogenic agent
- ☐ I am not sure

14. Which factors increase the potency of bisphosphonates? (Select all that apply.)

- ☐ Intravenous formulation
- ☐ Number of radical groups in the chemical structure
- ☐ Nitrogen content in the chemical structure
- ☐ Tablet (oral) formulation
- ☐ I do not know

15. Compared with intravenous administration, does routine oral use of bisphosphonates carry a higher risk of developing MRONJ?

- ☐ Yes
- ☐ No

16. Which of the following are considered risk factors for MRONJ? (Select all that apply.)

- ☐ Poor oral hygiene
- ☐ Periodontal disease
- ☐ Tobacco use
- ☐ Corticosteroid therapy
- ☐ Hypertension
- ☐ Hyperlipidemia
- ☐ Alcohol consumption

D. Questions Regarding the Dental Management of Patients with MRONJ

17. Which findings should be carefully evaluated during the clinical and radiographic examination of patients with MRONJ? (Select all that apply.)

- ☐ Halitosis
- ☐ Mucosal ulceration/inflammation
- ☐ Tooth mobility
- ☐ Exposed bone
- ☐ Thickening of the lamina dura
- ☐ Widening of the periodontal ligament space

18. Does your routine medical history form include the question: "Are you currently using or have you previously used antiresorptive or antiangiogenic medications?"

- ☐ Yes
- ☐ No

19. A 47-year-old female patient who has been taking oral alendronic acid for osteoporosis for one year and has no other systemic disease presents for tooth extraction. Would you perform the surgical procedure?

- ☐ Yes
- ☐ No

20. If you decide to perform the surgical procedure, would you implement any additional measures? (Follow-up to Question 19)

- ☐ Yes
- ☐ No

21. Which additional measures would you consider? (Select all that apply.)

- ☐ Preoperative antibiotic prophylaxis
- ☐ Postoperative antibiotic prophylaxis
- ☐ Consultation with the physician who prescribed the medication
- ☐ Assessment of the serum CTX level before surgery
- ☐ Other

22. In your opinion, what serum CTX level should be considered acceptable to minimize the risk of osteonecrosis during surgical procedures?

- ☐ <100 pg/mL
- ☐ 100–125 pg/mL
- ☐ 126–149 pg/mL
- ☐ >149 pg/mL

23. How would you approach a patient receiving antiresorptive or antiangiogenic therapy? (Select all that apply.)

- ☐ I would not provide treatment.
- ☐ I would provide only non-surgical treatment.
- ☐ I would perform minor surgical procedures (e.g., tooth extraction).
- ☐ I would perform surgical procedures (e.g., implant placement).
- ☐ I would provide all necessary treatments based on medical consultation.

24. Before tooth extraction or implant placement, for how long would you recommend discontinuing anti-osteoporotic medication?

- ☐ ≤2 months
- ☐ 3–5 months
- ☐ 6 months

25. How would you plan dental treatment before initiating intravenous bisphosphonate therapy? (Select all that apply.)

- ☐ Endodontic treatment for teeth with apical lesions
- ☐ Endodontic treatment for teeth with deep carious lesions
- ☐ Extraction of teeth with deep periodontal pockets
- ☐ Extraction of retained root fragments
- ☐ Monitoring of impacted teeth
- ☐ Extraction of teeth likely to cause pericoronitis
- ☐ I do not know
